# Labor market sorting and the gender pay gap revisited

**DOI:** 10.1007/s00148-025-01115-1

**Published:** 2025-07-08

**Authors:** Anthony Strittmatter, Conny Wunsch

**Affiliations:** 1 UniDistance Suisse, Brig, Switzerland; 2 Faculty of Business and Economics, University of Basel, Basel, Switzerland

**Keywords:** Gender inequality, Gender pay gap, Common support, Model specification, Matching estimator, Machine learning, J31, J71, C52, C55

## Abstract

This paper shows that gender segregation in the labor market has important implications for the estimation of gender pay gaps. Using Switzerland as an example, we provide evidence that there are sizable segments in the labor market with perfect sorting such that there are no comparable men and women. In these segments, covariate-adjusted gender pay gaps are not identified non-parametrically. Reliability of estimated pay gaps then requires correct functional forms for extrapolation or excluding segments of the labor market with perfect sorting from the analysis. We discuss different estimation choices within this trade-off and show how they affect estimates of unexplained gender pay gaps. We find that enforcing comparability ex ante, estimator choice and functional form restrictions matter greatly. Using a flexible semi-parametric estimator with moderate restrictions on ex ante comparability explains up to 38% more of the raw gender pay gap and results in estimated unexplained gender pay gaps that are up to 44% smaller than standard Blinder-Oaxaca estimates that account for the same wage determinants but ignore lack of overlap.

**Supplementary Information:**

The online version contains supplementary material available at 10.1007/s00148-025-01115-1.

## Introduction

Achieving gender equality in pay is among the top priorities for governments in many countries. Measuring gender inequality in pay, the so-called gender pay gap, has been the subject of extensive literature for more than half a century (see Blau and Kahn 2000; Goldin and Mitchell 2017, for comprehensive reviews).

A large part of this literature focuses on understanding the sources of inequality in pay by estimating how much of the gender pay gap can be explained by different characteristics and choices of men and women, and which part remains unexplained. These estimates serve as key inputs for policy makers’ decisions on measures for combating gender inequality in pay. One key insight from this literature is that sorting of men and women into different types of jobs is strong and explains a considerable part of the gender pay gap (e.g., Bayard et al. 2003; Goldin et al. 2017; Blau and Winkler 2021).

To estimate unexplained gender pay gaps, it would be ideal to compare men and women who are identical in all relevant wage determinants (Ñopo 2008). Strong sorting increases the risk that there are job segments for which there are no comparable men and women. For example, it will be difficult to find workers of the opposite gender who are comparable in all relevant dimensions in male-dominated sectors like construction or in female-dominated sectors like child care. In fully segregated job segments, a direct comparison of men and women is not possible because there is no overlap in the covariate distributions of men and women, also known as lack of common support. In this case, reliability of estimated gender pay gaps requires correct functional forms that allow extrapolating the relationship between wages and their determinants into regions without overlap. Alternatively, fully segregated job segments have to be excluded from the analysis, which affects both the size and the representativeness of the remaining sample.

In this paper, we investigate how labor market sorting and the resulting lack of common support affect gender pay gap estimates under different estimation choices. The objective of the paper is three-fold. Firstly, we document how severe common support violations are in samples of different sizes and how this affects the usefulness of the approach proposed by Ñopo (2008). Secondly, we show how sensitive estimates of unexplained gender pay gaps are to alternative estimation choices. Finally, we derive recommendations for applied researchers who aim to provide policy makers with estimates of explained and unexplained gender pay gaps that can serve as reliable inputs for their decision making.

We conduct the empirical analysis using Swiss data. Switzerland is an interesting case to study for two reasons. Firstly, it shares important features of both typically European and US-type labor markets. Switzerland has generous social insurance systems like many other European countries. At the same time, it has a very flexible labor market that is much less regulated than that of other European countries, which makes it more comparable to countries like the US. Secondly, Switzerland offers unique data. The Swiss Earnings Structure Survey that we use covers about 37,000 establishments of firms with individual data on more than 1.7 million employees in Switzerland. It contains an accurate measure of wages from the firms’ payment system and a rich set of observed individual, job and firm-level characteristics that may explain gender inequality in pay. Most importantly, the data cover almost one-third of all Swiss employees. They offer us sufficient degrees of freedom to study gender sorting in the labor market in detail and to compare a rich set of state-of-the-art estimation choices.

We start with implementing the approach of Ñopo (2008) to document the extent of support violations in our data and to provide a non-parametrically estimated benchmark for the unexplained gender pay gap in the subsample with support. Thereafter, we consider estimation choices that vary along three dimensions. Firstly, we implement five variants of common support enforcement with increasingly stricter overlap requirements. Secondly, we vary how flexibly we control for the observed wage determinants, which relaxes functional form assumptions for a given estimator. The baseline model contains dummy variables for all values of the categorical variables and quadratic terms for the continuous variables. A much more flexible alternative adds higher-order polynomials as well as a large number of interactions between wage determinants. Thirdly, we apply six different parametric and semi-parametric estimators that offer alternative ways to relax functional forms. As parametric estimators, we consider a linear regression model (LRM) with a dummy for women, and the Blinder-Oaxaca decomposition (BO) as the work-horse approach in applied work. As semi-parametric estimators, we include inverse probability weighting (IPW), augmented IPW (AIPW) as a doubly robust mixture between BO and IPW, and propensity score matching (PSM). Additionally, we propose a combination of exact matching and PSM (EXPSM) that aims at bringing PSM closer to the ideal of exact matching.

In total, we consider five definitions of common support, six estimators, and two model specifications for each estimator. This results in a total of 60 different estimates. By comparing these estimates, we make the involved trade-offs transparent and show how sensitive estimates are to these methodological choices. In the final step of the paper, we repeat our analyses in subsamples of considerably smaller size, as lack of common support is likely much more severe in smaller samples. Specifically, we draw random subsamples with 100,000 and 10,000 observations, respectively, and compare the results to the full sample with more than 1 million observations. Given the richness of our data, there is a high risk of overfitting in the sample with 10,000 observations, which could lead to imprecise estimates of the gender pay gap. To mitigate this risk, we incorporate machine learning (ML) methods among the estimation strategies, following a principled, data-driven approach to avoid overfitting.

We document the following findings. First, we observe strong sorting in the Swiss labor market. Female shares in ISCO 2-digit occupation groups range from below 5% to almost 90%. Within these groups, up to 28% of women and 58% of men have no comparable counterpart of the opposite gender in terms of broad groups of important wage determinants. Second, we show that the extent of support violations limits the usefulness of the exact matching approach of Ñopo (2008) even in very large datasets when more than only a few wage determinants are taken into account. Third, we find that all alternative estimation choices we consider significantly affect how much of the raw gender pay gap can be explained and how much remains unexplained with the same set of wage determinants. Compared to standard BO without support enforcement, estimates of the unexplained gender pay gap decline by up to 50%, while the explained part of the raw gap increases by up to 43%. Fourth, with estimates that are up to 39% lower, enforcing support has the strongest effect for all estimators. Fifth, flexible inclusion of wage determinants is very important for the parametric estimators LRM and BO, as well as the hybrid AIPW. In contrast, model specification has little impact on the results from semi-parametric matching estimators that do not model the wage equation. Sixth and finally, even with the most flexible model specification and a reasonable choice of common support, EXPSM reduces the estimated unexplained wage gap by as much as 20% relative to BO.

Based on our findings, we derive the following recommendations for applied researchers. Firstly, we advise checking common support ex ante and enforcing it moderately with respect to the most important wage determinants combined with robustness checks concerning how different support definitions affect results. Secondly, we recommend controlling for wage determinants in a flexible way for any choice of estimator. Thirdly, if samples are sufficiently large, we recommend using a semi-parametric estimator that combines exact matching on some important wage determinants with radius matching on the propensity score. This minimizes the risk of misspecification of the functional form and offers a reasonable balance between comparability, precision of the estimate, and representativeness of the study sample. Implementing this recommendation with our data explains 11% more of the raw wage gap sector than standard BO estimates and results in estimated unexplained pay gaps that are 16% lower.

The paper proceeds as follows. The next section discusses the related literature and details what we add. Section 3 describes our data and provides descriptive evidence on gender sorting in the labor market. Section 4 presents the econometric model. We implement exact matching on increasing sets of wage determinants and document the resulting support violations and non-parametric estimates of unexplained gender pay gaps. Section 5 discusses trade-offs in estimator choice and details the variants we consider. Section 6 presents the results and recommendations for applied research. The last section concludes. An Online Appendix contains supplementary material.

## Literature

This study contributes to the literature investigating the role of methodological choices in the analysis of the gender pay gap. A number of existing studies examine the implications of labor market sorting and resulting violations of common support for the estimation of gender wage differentials (e.g., Ñopo 2008). As shown in Table 1, these studies often rely on data sets containing between 50,000 and 300,000 observations and account for a relatively limited number of wage determinants, often ranging from 10 to 50 variables. Both the sample size and the dimensionality of wage determinants are critical factors influencing the likelihood and severity of common support violations.

In recent years, applied research has increasingly made use of large-scale administrative or survey data, such as the European Union Structure of Earnings Survey (SES), the Current Population Survey (CPS), the US Census, and the American Community Survey (ACS) (e.g., Bach et al. 2024; Goldin et al. 2017). These modern data sets generally comprise more than 1 million observations and include over 50 wage determinants. Despite their growing use, the extent to which common support violations affect gender pay gap estimates in such large and rich data environments remains largely unexplored.

**Table 1:** Literature overview: empirical setting

	Commmon	Sample	Number	Model
	suppport	size	of wage	flexibility
			determinants	analyzed
Black et al. (2008)	Yes	Medium	Many	No
Bonaccolto-Töpfer and Briel (2022)	Yes	Small	Very many	w/ ML
Djurdjevic and Radyakin (2007)	Yes	Medium	Many	No
Frölich (2007b)	No	Small	Many	w/o ML
Goraus et al. (2017)	Yes	Medium	Many	w/o ML
Meara et al. (2020)	Yes	Medium	Many	No
Ñopo (2008)	Yes	Large	Few	No
Current study	Yes	Very large	Very many	w/ ML

Notes: Sample size categories are defined as small (<50,000 observations), medium (50,000–300,000), large (300,000–1 million), and very large (>1 million). Control variable categories are defined as few (<10 variables), many (10–50 variables), and very many (>50 variables). “w/ ML” indicates that model flexibility is analyzed using machine learning techniques, whereas “w/o ML” refers to model flexibility analyzed using traditional model specification without the use of machine learning

The current study addresses this gap by being the first to systematically analyze common support violations in a data set comprising more than one million observations and more than 100 wage determinants. We are also the first to investigate the role of model flexibility in a data environment of this scale and richness. Moreover, we extend our analysis by simulating how the results would change in smaller sample scenarios, while holding the data structure constant. For this reason, we employ both manual, discretionary modeling decisions and data-driven approaches based on ML techniques, which are particularly promising for mitigating overfitting in smaller samples and enhancing estimation robustness. This allows us to provide novel insights into the trade-offs between sample size, model complexity, and the robustness of gender pay gap estimates under varying degrees of common support.

**Table 2:** Literature overview: estimation methods

	Estimation methods					
	LRM	BO	IPW/	EXM	PSM	EX-
Analyzed			AIPW			PSM
Black et al. (2008)	X	X		X		
Bonaccolto-Töpfer and Briel (2022)	X					
Djurdjevic and Radyakin (2007)		X		X	X	
Frölich (2007b)					X	
Goraus et al. (2017)	X	X		X	X*	
Meara et al. (2020)			X	X	X	
Ñopo (2008)		X		X		
Current study	X	X	X	X	X	X

Notes: * Goraus et al. (2017) apply an equivalent methodology for estimating quantile treatment effects based on DiNardo et al. (1996)

A further focus of our study is the choice of estimator, as summarized in Table 2. Commonly used approaches include the linear regression model (LRM) and the Blinder-Oaxaca (BO) decomposition. Ñopo (2008) argues that exact matching (EXM) is better suited to estimate gender pay gaps, as it ensures that only men and women with identical wage determinants are compared. However, a major drawback of EXM is the curse of dimensionality. The more wage determinants are taken into account, the more the common support may deteriorate. To overcome this limitation, several studies have proposed alternative semi-parametric estimators such as propensity score matching (PSM) and (augmented) inverse probability weighting (IPW/AIPW) (e.g., Djurdjevic and Radyakin 2007; Frölich 2007b; Goraus et al. 2017; Meara et al. 2020).

While propensity score methods mitigate the curse of dimensionality, they blur the comparability requirement: having the same propensity score implies neither identical wage determinants nor full support. We propose a hybrid estimator that balances the trade-off between exact matching and broader support. Specifically, we introduce exact propensity score matching (EXPSM), which enforces exact matching on a small set of the most important wage determinants and accounts for differences in the remaining covariates via the propensity score.

We are also the first to jointly evaluate commonly used examples of all major types of estimators—parametric, semi-parametric, and non-parametric—within a data environment that is both large and rich. By only varying one dimension at a time but looking at a large set of possible cases, we systematically investigate how common support violations affect gender pay gap estimates across estimators, model specifications and samples of different sizes, thereby offering a comprehensive understanding of the methodological trade-offs involved in applied research on gender wage inequality.

Our study also connects to broader strands of literature that assess the sensitivity of gender pay gap estimates to other dimensions. A large literature identifies wage determinants that contribute to explaining the gender pay gap and analyzes how including these determinants changes estimates of the gender pay gap (see Blau and Kahn 2000; Weichselbaumer and Winter-Ebmer 2005; Van der Velde et al. 2015; Olivetti and Petrongolo 2016; Goldin and Mitchell 2017; Blau and Kahn 2017; Kunze 2018, for reviews). A recent example is Casarico and Lattanzio (2023), who show that the child penalty significantly influences both wages and post-childbirth labor market sorting of mothers. Another strand of literature focuses on the assumptions required for the identification of gender discrimination. For example, there are studies that investigate biases due to gender-specific selection into employment (e.g., Chernozhukov et al. 2020; Machado 2017) and potential endogeneity of the observed wage determinants (e.g., Huber 2015; Yamaguchi 2015).

All our estimates take individual choices regarding employment and wage determinants as given. They rely on the same set of wage determinants and build on the same assumptions for non-parametric identification. The parametric and semi-parametric estimators we consider impose different additional assumptions that restrict functional form relationships, which are not necessary for non-parametric identification. Moreover, sample restrictions to enforce common support affect sample size and composition. The differences in our estimates result from differences in these functional form and support restrictions.

## Data

### Swiss earnings structure survey

We use the 2016 wave of the Swiss Earnings Structure Survey (ESS).^1^ The ESS is a bi-annual survey of approximately 37,000 private and public establishments with individual data on more than 1.7 million employees, representing almost one-third of all employees in Switzerland. The survey covers salaried jobs in the secondary and tertiary sectors in establishments with at least three employees. Sampling is random within strata defined by establishment size, industry, and geographic location. Participation in the survey is compulsory for the establishments. The gross response rate is higher than 80%.^2^ Typically, establishments report the required information directly from their remuneration systems. Thus, the survey effectively includes administrative data from establishments.

#### Measurement of wages

The main variable of interest is a standardized wage measure that is provided by the Federal Statistical Office as part of the ESS. It measures the monthly full-time-equivalent gross wage including extra payments. The latter comprise add-ons for shift, Sunday, night work, other non-standard working conditions and irregular payments such as bonuses and Christmas or holiday salaries, but they exclude overtime premia. Wages are standardized to a 100% full-time equivalent without overtime hours.

#### Observed wage determinants

The data contain a rich set of wage determinants that are considered important in the gender pay gap literature such as age, education, occupation, industry, establishment characteristics, wage bargaining, management level, marital status, and part-time work.^3^ But some important variables are not included in the data. One example is actual work experience (e.g., Cook et al. 2021). Potential experience is captured by age and education, and we also observe tenure. Hence, we capture some aspects of experience, but not all of it. Since experience is positively related to wages, and women have on average less work experience, we over-estimate potential violations of equal pay for equal work in Switzerland in this respect. However, other information that the literature emphasizes is missing as well. This includes, for example, competitiveness, children and other dependent household members, environment during childhood, gender norms, and non-cognitive factors.^4^ Moreover, we do not account for selection into employment based on unobserved characteristics or for potential endogeneity of control variables. Hence, our estimates are informative about equal pay for equal work when individual choices are taken as a given, subject to any omitted variable bias that may result from unobserved factors. Table A.1 in Supplementary material A provides a detailed description of all observed variables.

#### Comparison with other data sets

Our data are very similar to those of the European Union Structure of Earnings Survey (SES). The SES provides harmonized data on earnings in EU member states, candidate countries, and European Free Trade Association (EFTA) countries. It includes all quantitatively important wage determinants.^5^ Moreover, like the Swiss data, actual work experience is not included. Studies for the US typically use survey data collected from individuals such as the Current Population Survey (CPS), the US Census, or the American Community Survey (ACS). Such surveys contain much richer information on individuals but potentially suffer from measurement errors in self-reported wages. However, the key wage determinants we observe are included as well, and most studies use a similar set of variables. In terms of sample size, all of the data sets are also very large, containing several hundred thousand observations.

### Sample restrictions

We restrict the analysis to the working population aged between 20 and 59 years (dropping 127,298 employees). We exclude employees for whom we observe very little support between men and women ex ante. This excludes 70,052 employees with less than 20% part-time employment, 2706 members of the armed forces or agricultural and forestry occupations,^6^ and 3025 employees from the agricultural, forestry, mining, and tobacco sectors.^7^ We analyze the gender pay gap separately for the private and public sector. The public sector offers more regulated wages and attracts a different selection of employees. For example, women are over-represented in the public sector at 56% but constitute only 43% of the private sector. Moreover, the public sector is much more homogeneous in terms of industries and occupations. For better exposition, we focus on the private sector in the main text and present the results for the public sector in the appendix. The baseline sample contains 1,132,042 employees in the private sector and 405,448 employees in the public sector, after dropping an additional 54 employees in industries with very few observations in the public sector.^8^

**Table 3:** Means and standardized differences: private sector

	Mean		Std
	Women	Men	Diff
	(1)	(2)	(3)
*Wage*			
Standardized monthly wage (in CHF)	6266	7793	31.9
*Demographics*			
Age	40.26	40.51	2.3
Education			
University	.14	.17	8.2
Vocational	.62	.62	0.3
No vocational	.19	.17	4.9
*Job characteristics*			
Part-time	.59	.15	103.4
Tenure	6.39	7.64	16.0
Management level			
Top	.03	.07	18.8
Upper	.05	.08	12.0
Middle	.08	.10	7.7
Lower	.07	.08	4.3
None	.77	.67	23.2
Irregular wage components (e.g., bonuses)	.33	.41	16.9
Occupation (ISCO 1-digit)			
Managers	.07	.12	18.8
Professionals	.13	.15	5.3
Technicians & Associate Professionals	.24	.20	11.3
Clerical Support Workers	.14	.05	32.0
Services & Sales Workers	.27	.11	42.5
Craft & Related Trades Workers	.03	.21	56.6
Plant & Machine Operators & Assemblers	.02	.09	31.9
Elementary Occupations	.10	.08	6.7
*Employer characteristics*			
Industry			
Low-tech manufacturing	.07	.14	21.1
High-tech manufacturing	.06	.11	15.4
Less knowledge-intensive services	.40	.32	18.3
Knowledge-intensive services	.44	.28	32.3
Other (incl. construction)	.03	.16	47.5
Firm size			
$\le$ 20	.26	.22	9.5
20–49	.13	.16	8.9
50–249	.24	.26	5.0
250–999	.14	.16	4.4
$\ge$ 1000	.24	.21	6.9
Observations	491,007	641,035	

Notes: This presents mean values by gender and the standardized differences (std. diff.) between women and men, based on the baseline sample prior to imposing any support restrictions. Monthly regular wages are standardized to 100% full-time equivalents and exclude overtime hours

### Descriptive evidence on gender sorting

To shed first light on sorting of women and men into different types of job, Table 3 documents the means and standardized differences of selected variables by gender for the private (and A.2 in Supplementary material A for the public sector). In the private sector, the average standardized monthly wage is 6266 CHF (1 CHF $\approx$ 1 USD in 2016) for women and 7793 CHF for men, implying a raw wage gap of 18.6%. In the public sector, the average wage is much higher at 7731 CHF for women and 8985 CHF for men resulting in a raw wage gap of 13.9%. The Swiss raw gap is very similar to the gaps observed in Germany and the UK, and only slightly smaller than the gap in the US (OECD 2021).

The distributions of age and education are relatively similar across gender in both sectors, although employed women tend to have slightly less education than men. Women have less tenure and are substantially more likely to work part-time than men. There is a large gender difference in the share of workers holding a management position. Considerably more managers are men and the standardized difference is particularly large for top managers. These differences are reflected in a sizeable gender gap in irregular wage components such as bonuses. Occupational sorting is strong as well. Clerical support, services, and sales occupations attract a much higher share of females than males, while craft and related trades occupations as well as plant and machine operators and assemblers are male-dominated. Sorting is also very strong with respect to industry. Considerably more men work in manufacturing than women. The difference is even larger for other industries, which include the construction sector. Gender differences are small with respect to firm size of the employer.^9^

**Fig. 1:**
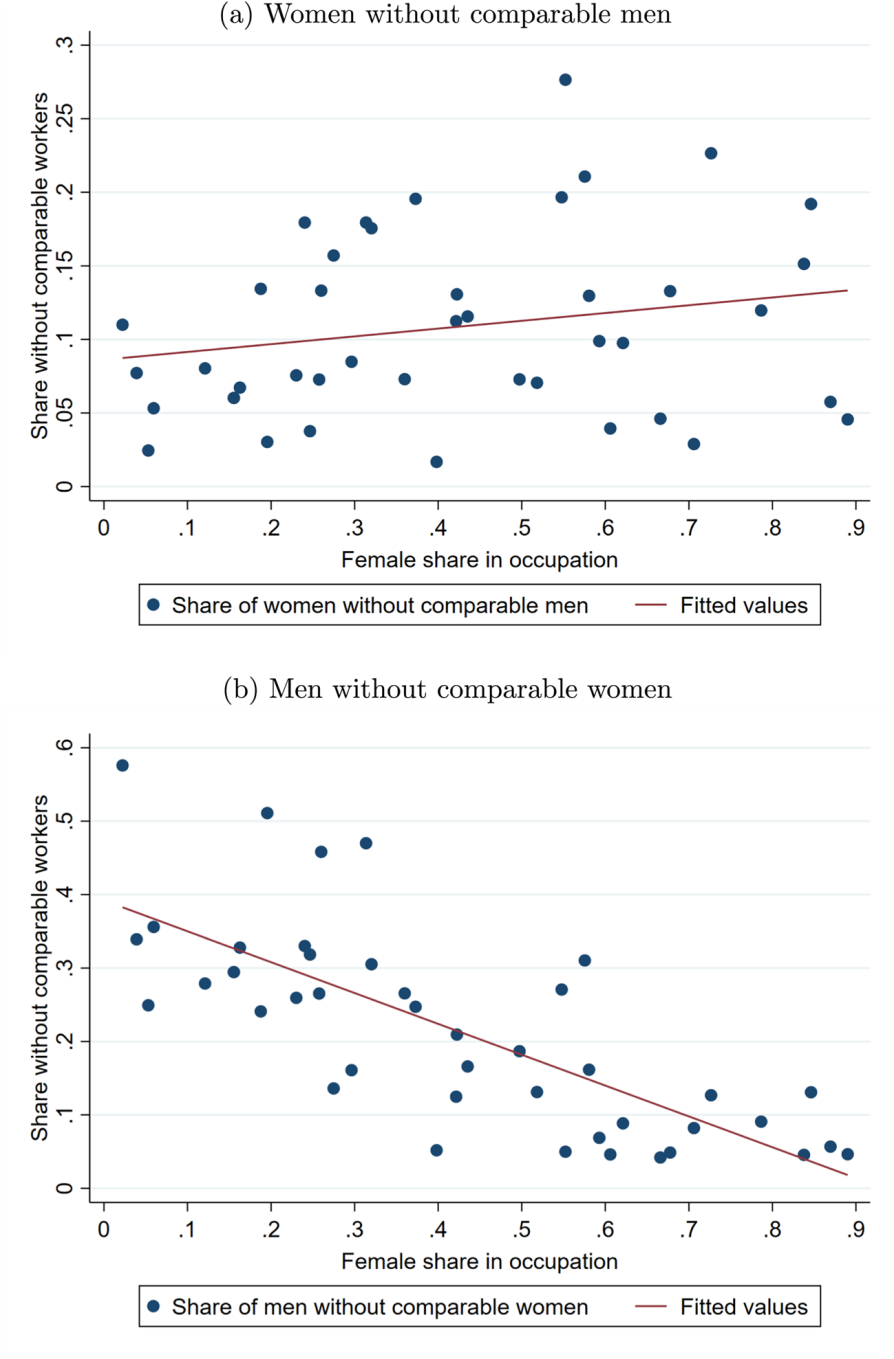
Lack of comparability within occupation groups. Notes: This displays the share of workers within occupations for whom there is no worker of the opposite gender who is comparable in terms of sector (private/public), education (5 groups), age (4 groups), tenure (5 groups), industry (15 groups), and management level (5 groups). The fitted values are based on a linear regression. Occupation groups are defined at the two-digit level of the International Standard Classification of Occupations (ISCO-08)

In Fig. 1, we take a closer look at gender sorting in the labor market. Firstly, it shows the share of females working in a given ISCO 2-digit occupation (42 groups). We find that occupational sorting is very strong with female shares in occupations ranging from well below 5% to almost 90%. Secondly, Fig. 1 displays the share of women and men within occupations for whom there is no comparable employee of the opposite gender in terms of broad groups of important wage determinants. These include sector (private/public), education (5 groups), age (4) and tenure (5) as proxies for experience, industry (15), and management level (5). We find that sorting is also strong within occupations. Up to 28% of women and up to 58% of men in an occupation work in job segments without comparable workers of the opposite gender. This is substantial given our very large data set and the broad groups we look at. As expected, lack of comparability increases with the female share for women and decreases for men. We also see that fully segregated job segments are more frequent for men in male-dominated occupations than for women in female-dominated occupations.

Overall, Fig. 1 provides compelling evidence that gender sorting in the labor market is strong and leads to lack of comparability for a sizeable fraction of female and male workers. In the next section, we investigate this issue further and discuss the implications for estimating covariate-adjusted gender pay gaps.

## Common support and the gender pay gap

### Econometric model and non-parametric identification

The raw gender pay gap is the difference between the average wages of women and men. Let $G_i$ denote the gender dummy, with $G_i=1$ for employed women and $G_i=0$ for employed men. Further, let $Y_i$ denote the logarithm of the standardized monthly wage. With this notation, the raw gender pay gap is

4.1 $$\begin{aligned} \Delta = E[Y_i|G_i=1] - E[Y_i|G_i=0]. \end{aligned}$$

When studying gender inequality in pay, researchers are typically interested in decomposing the raw gender pay gap (4.1) in the part that can be explained by observed wage determinants $X_i$ and the remaining unexplained part. Let $E_{X|G=1}[\mu _0(x)]$ denote the predicted wage of employed men, had they had the same observed characteristics as employed women, with $\mu _0(x) = E[Y_i|G_i=0,X_i=x]$. Adding and subtracting $E_{X|G=1}[ \mu _0(x) ]$ in Eq. 4.1 gives

4.2 $$\begin{aligned} \Delta = \underbrace{ E[Y_i|G_i=1] - E_{X|G=1}[ \mu _0(x) ]}_{\text{ unexplained } \delta } +\underbrace{ E_{X|G=1} [ \mu _0(x) ] - E[Y_i|G_i=0]}_{\text{ explained } \eta }. \end{aligned}$$

The second term on the right-hand side of Eq. 4.3, $\eta$, is the part of the raw pay gap that can be explained by gender differences in the observed wage determinants $X_i$ using the wage of males as benchmark. The first term on the right-hand side of Eq. 4.3, $\delta$, is the gender pay gap for employed women that cannot be explained by these differences. It is the expected difference in pay of employed women compared to observationally identical employed men, which is the parameter of interest in the majority of studies on gender wage inequality.^10^

Without further assumptions, an empirical counterpart for $E_{X|G=1}[ \mu _0(x) ]$ exists only if for each woman there is at least one man who is observationally identical with respect to the wage determinants $X_i$. Formally, this corresponds to the requirement that there is common support in the distributions of covariates $X_i$ for men and women^11^:

4.3 $$\begin{aligned} 0\le Pr(G_i=1|X_i=x)<1. \end{aligned}$$

Violation of common support is more likely the stronger men and women with distinct characteristics sort into specific jobs. For example, in female-dominated occupations like child care or in male-dominated occupations in construction, it will be difficult to find comparable of the opposite gender as shown in Fig. 1. Let $S_{i}=1$ for individuals with support and $S_i=0$ for individuals without support. Ñopo (2008) shows that

4.4 $$\begin{aligned} \Delta =E[\delta +\eta |S_i=1] +\rho . \end{aligned}$$

The term $E[\delta +\eta |S_i=1]$ is the raw pay gap within support, which can be decomposed into the parts explained ($\eta$) and unexplained ($\delta$) by differences in *X*. The last term $\rho \equiv \rho _1-\rho _0$ is the part of the raw wage gap that is due to lack of support, where $\rho _g\equiv Pr(S_i=0|G_i=g)[E[Y_i|G_i=g,S_i=0]-E[Y_i|G_i=g,S_i=1]]$ measures the wage difference across individuals of gender $g\in \{0,1\}$ out of and within support.

**Fig. 2:**
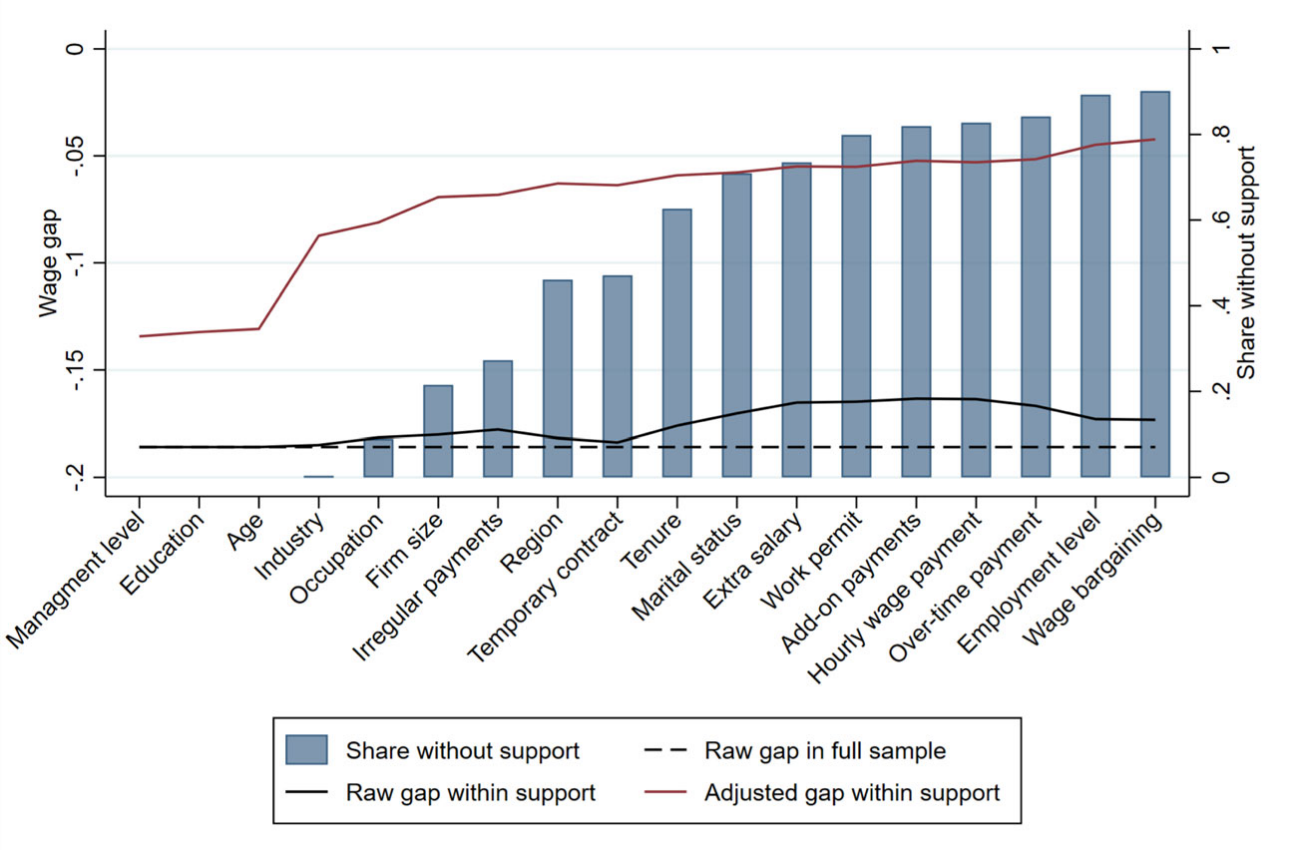
Common support and unexplained pay gaps from exact matching in the private sector. Notes: The indicated wage determinants are added sequentially from left to right. The raw gap within support refers to the difference between the average log wages of women and men in the sample with support, considering all wage determinants from the left up to the respective determinant. The unexplained gap is obtained from exact matching on all wage determinants from the left up to the respective determinant

### Exact matching and the curse of dimensionality

Nonparametric approaches for estimating $E_{X|G=1}[ \mu _0(x) ]$ do not impose any assumptions on the underlying functional forms. Exact matching (EXM) as used, for example, by Ñopo (2008), is one of such approaches. It stratifies the sample into $K\ll N$ mutually exclusive groups $W_i\in \{1,..., K\}$, which are defined based on the characteristics $X_i$. Then, it estimates $E_{X|G=1}[ \mu _0(x) ]$ as the strata-specific average wages weighted by their observed shares among females:

4.5 $$\begin{aligned} \frac{1}{N_1} \sum _{i=1}^{N} G_i \left( \sum _{j=1}^{N} \frac{\displaystyle 1\{W_i= W_j\} (1-G_j)Y_j}{\displaystyle \sum _{j=1}^{N} 1\{W_i= W_j\} (1- G_j)} \right) . \end{aligned}$$

When studying gender inequality in pay, one would ideally account for gender differences with respect to all observed wage determinants. However, the ability to do so without any parametric assumptions is limited by the curse of dimensionality. The more strata there are, the more likely it is that a given stratum is empty for males but not females. While this is true mechanically in any given sample, the problem is more severe the stronger men and women sort into different types of jobs.

In Fig. 2, we systematically increase the number of strata we use to enforce common support and to estimate the unexplained gender pay gap with EXM. Specifically, we increase the number of variables on which we match exactly. We add variables based on their importance for predicting counterfactual male wages. For this purpose, we run a simple wage regression in the sample of men (i.e., we estimate $\mu _0(x)$). We measure importance as the change in adjusted $R^2$ when we omit one block of variables (e.g., all occupation dummies), but keep all other wage determinants. We sort all variable blocks in decreasing order according to the $R^2$ changes. We report the resulting order of variables and the corresponding changes in the adjusted $R^2$ as well as the full results of the Ñopo (2008) decomposition (4.4) in Table B.1 in Supplementary material B.^12^

#### Common support

We find that there is full support with respect to the three most important wage determinants: management level, education, and age. The loss of observations due to lack of support is relatively small when we add industry and occupation, which are both highly relevant for predicting wages. It becomes larger but remains below 20% if we add firm size and a dummy for irregular payments. Thereafter, however, support deteriorates quickly. Once we enforce support with respect to the 10 variables that are most important for explaining male wages, 55% of women in the private sector have no comparable men with respect to these variables. This shows that gender segregation in the labor market in substantial. Subsequently adding the less important wage determinants reduces common support further. When enforcing full support with respect to all observed wage determinants, 89% of women in the private sector have no comparable men. The massive loss of observations due to lack of support shows that exact matching as proposed by Ñopo (2008) comes at a very high cost. It reduces precision of the estimates considerably and strongly affects the representativeness of the remaining sample. Therefore, we discuss alternative estimation choices in the next section.^13^

#### Raw gender pay gaps

Figure 2 further shows the raw gender pay gaps in the different samples with common support. Additionally, Table B.1 in Supplementary material B reports the part $\rho$ of the raw wage gap that is due to lack of support. The raw gap in the private sector is relatively insensitive to the support definition. It decreases only slightly, from 18.6% under the weakest support definition to 17.3% under the strongest one. The maximum value of $\rho$ is 0.022, which corresponds to 12% of the raw gap.^14^ Our results are consistent with Ñopo (2008), who finds that 11% of Peru’s gender pay gap estimates can be explained by lack of common support regarding age, education, marital status, and migration condition.

#### Unexplained gender pay gaps

Finally, Fig. 2 shows the relationship between including more variables in the exact matching procedure and the resulting unexplained gender pay gap for the women with support. As expected, the unexplained gender pay gaps decrease when we add more variables. They fall from 13.4% under the weakest support definition to 4.2% under the strongest one.^15^ It is important to keep in mind, though, that the composition of the samples becomes increasingly different with stricter support enforcement when matching on more variables.

## Trade-offs in estimator choice

### Parametric estimators

Parametric approaches for estimating explained and unexplained wage gaps assume a specific functional form for $\mu _0(x)$ and $\mu _1(x)$. Enforcing common support is not necessary because they extrapolate into regions without support based on the assumed functional form. However, they are biased and lead to different results than exact matching if this functional form is not correct. The two most common parametric estimators for the gender pay gap are the linear regression model and the Blinder-Oaxaca decomposition.

#### Linear regression model (LRM)

A simple estimation approach for the unexplained gender pay gap employs a linear regression model with a dummy for women,

5.6 $$\begin{aligned} Y_i = \alpha + G_i {\delta }_{LRM} + X_i{\beta } + {\varepsilon }_i, \end{aligned}$$

where $\alpha$ is a constant, the vector ${\beta }$ describes the association between the wage determinants $X_i$ and the wages, and ${\varepsilon }_i$ is an error term. The parameter ${\delta }_{LRM}$ can be interpreted as the unexplained gender pay gap. The LRM assumes that $\mu _0(x)=\alpha +X_i{\beta }$ and that $\mu _1(x)=\mu _0(x)+\delta _{LRM}$. Thus, it imposes homogeneous coefficients for the wage determinants and assumes that the unexplained gender pay gap is equal for women and men. This assumption is unnecessarily restrictive, because it can be relaxed by including interaction terms between gender and the wage determinants.

#### Blinder-Oaxaca decomposition (BO)

Originating from Blinder (1973) and Oaxaca (1973), BO relaxes the functional form assumptions for $\mu _0(x)$ and $\mu _1(x)$ by allowing for gender differences in the impact of characteristics $X_i$ on wages as well as for heterogeneity in the unexplained gender pay gap that is driven by these differences. It assumes that $\mu _g(x)=\alpha _g+X_i{\beta _g}$ for $g\in \{0,1\}$, implying that $\delta _{BO}=(\alpha _1-\alpha _0)+X_i(\beta _1-\beta _0)$. We implement BO as a two-step estimator. In the first step, we estimate in the subsample of men the linear wage model

5.7 $$\begin{aligned} Y_i = \alpha _0 + X_i{\beta }_0 + {u}_i, \end{aligned}$$

where $\alpha _0$ is an intercept and ${u}_i$ is an error term. The coefficients ${\beta }_0$ describe the association between the wage determinants $X_i$ and the wages of men. In the second step, we use the estimated coefficients from this regression (indicated by hats) to predict the counterfactual male wage $\hat{\mu }_0(X_i)\equiv \hat{\alpha }_0 + X_i\hat{\beta }_0$ for each woman in the sample and estimate the mean unexplained gender pay gap for women using

5.8 $$\begin{aligned} \hat{\delta }_{BO} = \frac{1}{N_1} \sum _{i=1}^{N} G_i (Y_i - \hat{\mu }_0(X_i) ) \end{aligned}$$

with $N_1 = \sum _{i=1}^{N} G_i$. The BO model corresponds to the LRM augmented with interaction terms between gender and all observable wage determinants:

5.9 $$\begin{aligned} Y_i = \alpha _0 + G_i \underbrace{(\alpha _1-\alpha _0)}_{=\alpha _{BO}} + X_i{\beta _0} + G_i X_i \underbrace{(\beta _1-\beta _0)}_{= \beta _{BO}} + {\epsilon }_i. \end{aligned}$$

Using this fully interacted LRM, we could estimate the BO unexplained wage gap by

$$\begin{aligned} \hat{\delta }_{BO} =\hat{\alpha }_{BO} + \frac{1}{N_1} \sum _{i=1}^{N} G_i X_i\hat{\beta }_{BO}. \end{aligned}$$

This estimation procedure is numerically identical to Eq. 5.8 when we control for the same characteristics $X_i$ as in Eq. 5.7.^16^

### Semiparametric estimators

Similar to non-parametric estimators, semi-parametric approaches do not assume a specific functional form for $\mu _0(x)$ or $\mu _1(x)$. They do not restrict the relationship between the wage and its determinants *X* and allow for arbitrary heterogeneity in unexplained wage gaps, thereby avoiding functional form misspecification. At the same time, they also avoid the curse of dimensionality because they do not compare men and women with respect to the high-dimensional vector of wage determinants $X_i$. Instead, they collapse all information about gender differences in $X_i$ in a single number, the so-called propensity score, which is the conditional on $X_i$ probability of being a woman:

5.10 $$\begin{aligned} {p}(X_i) = {Pr}(G_i=1|X_i) = F( X_i{\gamma } ). \end{aligned}$$

$F(\cdot )$ is a binary link function that is typically estimated parametrically. After estimating this model, semi-parametric approaches construct weights $\hat{W}_i^{0}$ based on the estimated propensity score $\hat{p}(X_i)$ that are used to reweigh the male observations such that their distribution of wage determinants $X_i$ resembles the one among females after re-weighting. While semi-parametric estimators also require common support (4.3), it can be enforced based on the one-dimensional propensity score rather than the high-dimensional vector $X_i$. This avoids the curse of dimensionality and alleviates support issues considerably.

#### Inverse probability weighting (IPW)

Based on ideas originating from Horvitz and Thompson (1952); Hirano et al. (2003), it shows that the following estimator appropriately removes group differences in observed characteristics $X_i$ if the underlying model for the propensity score is specified correctly:

5.11 $$\begin{aligned} \hat{\delta }_{IPW} = \frac{1}{N_1} \sum _{i=1}^{N} G_i Y_i - \sum _{i=1}^{N}\frac{(1-G_i)\hat{W}_i^{0} Y_i}{\sum _{i=1}^{N} (1-G_i)\hat{W}_i^{0}},\ \text {with}\ \hat{W}_i^{0} = \displaystyle \frac{\hat{p}(X_i)}{1-\hat{p}(X_i)}. \end{aligned}$$

The final weights are normalized such that they add up to one in finite samples (see, e.g., Busso et al. 2014). In contrast to LRM and BO, IPW does not impose any specific functional form on the relationship between $X_i$ and the wage, and, as a result, it does not restrict heterogeneity in unexplained gender pay gaps. A potential disadvantage of IPW is its instability when the conditional probability of being a woman is close to one, which may result in a high variance of $\hat{\delta }_{IPW}$. We impose trimming rules to avoid propensity score values that are too extreme (see the discussion in, e.g., Lechner and Strittmatter 2019). In particular, we omit males with weights $\hat{W}_i^{0}$ above the 99.5% quantile. We document the number of trimmed observations in Table B.4 of Supplementary material B.

Kline (2011) argues that BO estimators are equivalent to propensity score re-weighting estimators when the implicit weights of BO are non-negative. However, in contrast to IPW, which typically uses Logit or Probit, BO uses a linear model specification. As a result, the implicit weights of BO can become negative in practice, which is not possible for the IPW estimator. However, in the limit to a fully saturated BO model, even the linear specification of the propensity score would be well behaved and negative weights would be unlikely. Accordingly, the more flexible the model specification, the more the estimation results of BO and IPW should converge.

#### Augmented inverse probability weighting (AIPW)

Dating back to Robins et al. (1994), AIPW has received significant attention since Chernozhukov et al. (2018) proposed this estimation procedure in the context of machine learning. AIPW is a doubly robust mixture between the BO and IPW approach. Let $\tilde{W}_i^{0}$ denote the normalized IPW weights. Then, the AIPW estimator is given by

5.12 $$\begin{aligned} \hat{\delta }_{AIPW} = \frac{1}{N_1} \sum _{i=1}^{N} G_i (Y_i - \hat{\mu }_0(X_i) ) - \sum _{i=1}^{N} \tilde{W}_i^{0} (Y_i - \hat{\mu }_0(X_i) ), \end{aligned}$$

with $\hat{\mu }_0(X_i)$ as in BO. The first right-hand term in Eq. 5.12 is equivalent to BO in Eq. 5.8. The second right-hand term in Eq. 5.12 has an expected value of zero but makes a finite sample adjustment by re-weighting any observed bias of $\hat{\mu }_0(X_i)$ in the sample of men with the IPW weights. As a result, AIPW is more robust to misspecification than BO or IPW. In particular, $\hat{\delta }_{AIPW}$ is consistent even when either $\hat{\mu }_0(x)$ or $\hat{p}(x)$ is misspecified. Moreover, the theoretical properties of AIPW are well established for generic estimators of the nuisance parameters $\hat{\mu }_0(x)$ and $\hat{p}(x)$.

In particular, $\root 4 \of {N}$-consistency of $\hat{\mu }_0(x)$ and $\hat{p}(x)$ is sufficient to achieve $\sqrt{N}$-consistency of $\hat{\delta }_{AIPW}$ (in combination with the cross-fitting procedure described below). This permits the application of flexible machine learning methods to estimate $\hat{\mu }_0(x)$ and $\hat{p}(x)$, which often have a slower convergence rate than $\sqrt{N}$. AIPW combined with machine learning is often called double-machine-learning.

#### Propensity score matching (PSM)

While IPW uses all male observations to construct the counterfactual male wage $E_{X|G=1}[ \mu _0(x) ]$, PSM estimators only use men that are sufficiently comparable in terms of the propensity score. There are different estimators that apply different criteria for finding appropriate comparisons. For example, nearest neighbor matching uses the most similar observation. This minimizes bias but reduces efficiency if many similar comparisons are available. Radius matching overcomes this drawback (see, e.g., Frölich 2007b; Lechner and Wunsch 2009; Lechner et al. 2011). It matches to each woman all men who have a propensity score within a certain absolute difference (radius) of the woman’s propensity score. Then, it weighs all men within the radius with a weight, that is inversely related to the absolute propensity score difference. We define the radius as the 99% quantile of the distribution of the closest absolute distances for all women (the nearest neighbors), and then omit women without a match within the radius. As weights, we use the inverse of the absolute propensity score difference. Table B.5 in Supplementary material B documents the remaining number of females. We only lose relatively few women who lack matching men within the defined radius.

#### Combining exact and propensity score matching (EXPSM)

We also consider mixtures between exact and propensity score radius matching as suggested, for example, in Rubin and Thomas (2000). Here, we exactly match on the wage determinants that define support, as in EXM, and apply propensity score radius matching within each stratum using all wage determinants, as in PSM. In contrast to EXM, the propensity score corrects semi-parametrically for remaining within strata observable differences between women and men. In contrast to PSM, we enforce exact comparability of women and men with respect to some wage determinants.^17^

#### Other estimators

There are a large number of alternative estimators that could be used to handle high-dimensional covariates. These include, for example, Kernel or Mahalanobis distance matching (Huber et al. 2013), non-parametric regressions (Frölich 2007a), entropy balancing (Hainmueller 2012), and approaches to improve covariate balancing based on the propensity score such as Imai and Ratkovic (2014). To keep the number of estimators we study tractable, we focus on IPW and PSM as the most commonly used semi-parametric estimators in applied work and add EXPSM as a hybrid between exact and semi-parametric matching that directly addresses common support in a transparent way.

### Model specification for a given estimator

Even for a given estimator, functional forms can be relaxed by including higher-order polynomials of continuous wage determinant and interaction terms between variables. In particular, more flexible specifications should reduce differences across estimators. In fact, if all wage determinants were discrete, a fully saturated model that includes dummies for all possible values and a full set of interactions would be equivalent to exact matching (Angrist and Pischke 2008). To assess the implications of model flexibility for estimating wage gaps, we consider three different model specifications for the wage equation ${\mu }_0(x)$ and the propensity score *p*(*x*) (so-called nuisance parameters): the baseline, full, and machine learning (ML) models.

#### Baseline model

In the baseline model, we control for all observed variables in a standard way. Specifically, we include age (linear and squared), tenure (linear and squared), vocational education (nine categories), citizen status (six categories), marital status (three categories), occupation (39 categories ), industry sector (36 categories (public) sector ), management level (five categories ), region (7 categories), establishment size (five categories), and employment level (four categories). Further, we control for the dummy variables of temporary employment, the employment contract with hourly wages, collective wage agreement, overtime hours payment, bonus payments (e.g., from profit sharing), supplementary wages (e.g., for shift work), and extra salary (e.g., Christmas and holiday salaries). Overall, the baseline model includes 117 control variables for the private sector.

#### Full model

In the full model, we add to the baseline model all non-linear and interaction terms that we think could potentially be relevant. The non-linear terms are the polynomials of age and tenure up to order seven, as well as four age and five tenure categories. In addition, we include interaction terms to allow for heterogeneous returns to important wage determinants. Specifically, we interact occupation and industry groups with the categorical variables for age, tenure, education, foreigner status, management level, and establishment size. We also include interaction terms between establishment size categories and the categorical variables for age, tenure, vocational education, migration, and management level. Finally, we interact vocational education and management level categories with age and tenure categories. Overall, the full model includes 615 variables for the private sector.

#### Machine learning model (ML)

The ML model selects the relevant wage determinants and model flexibility in a data-driven way, using the full model specification as input. In practice, it is often unclear which non-linear and interaction terms are relevant, and we face a bias-variance trade-off. Using a too parsimonious model can bias ${\delta }$ due to model misspecification. Allowing for too much flexibility can lead to imprecise estimates of ${\delta }$ with high variance, especially in smaller samples. ML models allow accounting for relevant wage determinants and optimal specification of functional forms in a data-driven way, balancing flexibility and parsimony without manual tuning. While ML approaches can be applied to large samples, they are particularly promising in smaller samples where the full model specification may suffer from overfitting. Consequently, we discuss ML results primarily in the context of small-sample simulation analyses in Sect. 6.5. We describe how we implemented the ML models in Supplementary material C.

### Enforcement of common support

To gain further insights on the consequences of gender-based sorting in the labor market for the estimation of unexplained wage gaps we vary how we enforce common support ex ante. We pick five definitions of support that illustrate the trade-off between comparability, sample size, and representativeness. Table 4 lists the variables we use in the different definitions, the share of women without support, and the remaining number of women.

**Table 4:** Definitions of common support

Support	Variables for enforcing common support	Share women	Sample
version		outside support	size
1	Management level, education, age	.00	491,007
2	+ industry, occupation, firm size	.15	447,258
3	+ irregular payments, region, temporary contract	.39	369,524
4	+ tenure	.55	294,275
5	+ all other observed variables	.89	87,749

Notes: This defines the five versions of common support used in the main analysis. Variables are added sequentially from top to bottom to enforce common support. The third column reports the share of women excluded from the sample due to lacking common support. The fourth column shows the remaining number of women in the sample after enforcing support at each step

**Table 5:** Average characteristics of women by support version in the private sector

	Mean	Mean	Std. diff	Mean	Std. diff	Mean	Std. diff	Mean	Std. diff.
Support	1	2	2 vs. 1	3	3 vs. 1	4	4 vs. 1	5	5 vs. 1
Standardized monthly wage (in CHF)	6534	6506	0.8	6501	1.0	6454	2.3	6457	2.1
Irregular payments (incl. bonuses)	0.397	0.398	0.3	0.398	0.2	0.393	0.9	0.434	7.5
Age	40.2	40.1	0.6	40.0	1.7	39.6	5.2	37.1	27.3
Education: university	0.145	0.140	1.5	0.135	3.0	0.132	3.7	0.137	2.2
Education: vocational	0.574	0.571	0.6	0.555	3.7	0.535	7.7	0.477	19.4
Education: no vocational	0.191	0.195	1.0	0.200	2.2	0.205	3.5	0.195	1.1
Tenure	7.2	7.3	1.0	7.5	3.8	7.6	4.6	8.0	9.5
Management level: top	0.015	0.012	2.5	0.009	6.4	0.006	9.6	0.004	11.4
Management level: upper	0.041	0.037	2.4	0.033	4.4	0.030	6.1	0.034	3.6
Management level: middle	0.068	0.061	2.8	0.056	5.0	0.052	7.0	0.051	7.5
Management level: lower	0.066	0.058	3.6	0.053	5.8	0.048	8.0	0.051	6.5
Management level: none	0.809	0.832	6.0	0.850	10.9	0.865	15.3	0.860	13.7
Occupation: managers	0.076	0.069	2.7	0.063	5.1	0.059	6.9	0.075	0.6
Occupation: professionals	0.132	0.130	0.6	0.125	2.0	0.117	4.3	0.112	6.2
Occupation: technicians and associate professionals	0.261	0.267	1.2	0.264	0.7	0.254	1.8	0.193	16.4
Occupation: clerical support workers	0.109	0.093	5.3	0.074	12.3	0.065	15.7	0.091	6.0
Occupation: services and sales workers	0.269	0.285	3.7	0.317	10.6	0.349	17.4	0.330	13.4
Occupation: craft and related trades workers	0.029	0.029	0.1	0.028	0.5	0.027	1.4	0.024	3.1
Occupation: plant/machine operators, Assemblers	0.022	0.023	0.8	0.023	0.6	0.021	1.3	0.022	0.2
Occupation: elementary occupations	0.101	0.103	0.8	0.105	1.4	0.108	2.5	0.153	15.7
Part-time	0.552	0.551	0.3	0.549	0.6	0.549	0.5	0.321	48.0
Industry: low-tech manufacturing	0.065	0.060	2.1	0.052	5.3	0.045	8.4	0.038	12.0
Industry: high-tech manufacturing	0.090	0.089	0.5	0.087	1.1	0.082	2.9	0.088	0.9
Industry: less knowledge-intensive services	0.377	0.384	1.6	0.402	5.3	0.425	9.8	0.528	30.9
Industry: knowledge-intensive services	0.454	0.455	0.1	0.449	1.0	0.440	2.8	0.340	23.5
Industry: other (incl. construction)	0.014	0.012	1.7	0.009	4.7	0.007	6.9	0.006	8.5
Firm size: $\le$20	0.074	0.057	6.7	0.032	18.7	0.021	25.2	0.009	32.9
Firm size: 20–49	0.065	0.054	4.7	0.036	13.5	0.023	20.5	0.012	28.1
Firm size: 50–249	0.247	0.248	0.1	0.232	3.6	0.210	8.8	0.114	35.1
Firm size: 250–999	0.203	0.205	0.5	0.205	0.5	0.194	2.2	0.153	13.1
Firm size: $\ge$1000	0.411	0.436	5.1	0.495	17.1	0.552	28.5	0.712	63.7
# Observed women	491,007	447,258		369,524		294,275		87,749	

Notes: This shows mean characteristics of women across support versions 1 to 5 (see Table 4). Standardized differences (Std. Diff.) are relative to support version 1. Monthly wages are standardized to 100% full-time equivalents and exclude overtime hours

Support 1 enforces ex ante comparability with respect to management level, educational attainment, and age. It does not change the sample and is equivalent to the case without support enforcement for the private sector. Support 2 enforces support with respect to classical and very important wage determinants. Specifically, it adds industry, occupation, and firm size. The corresponding loss of observations is moderate with 15%. Support 3 covers all quantitatively important wage determinants by adding indicator variables for irregular payments and temporary contracts as well as region. This results in a sizeable share of 39% of women without support in the private sector. Support 4 adds tenure as the only proxy for actual experience, but it does not significantly explain wages. This substantially increases lack of support to 55%. Support 5 is the most extreme case, which we include for completeness rather than as a realistic scenario. It enforces full support with respect to all observed wage determinants and excludes 89% of the original sample of women in the private sector

Table 5 shows how stricter support enforcement affects the average characteristics of the remaining women with support, as well as the standardized differences to Support 1.^18^ Compositional differences across estimation samples with different ex ante support enforcement are important for the interpretation of our results. In the presence of heterogeneous pay gaps, estimates may differ not only because of methodological choices but also because the sample composition changes. This concerns differences in both observed wage determinants and unobserved factors, such as having small children that are correlated with observed factors like part-time work and tenure. We find noticeable changes in sample composition for only a few variables starting with Support 3. The most significant differences arise in Support 5 with respect to age, part-time work, industry, and firm size. Standardized differences exceed 20 for some characteristics only when Supports 4 and 5 are imposed. Overall, while there are some compositional shifts that may affect the results, they are modest, particularly under Support 2 and Support 3.

## Results

### Unexplained gender pay gaps

**Fig. 3:**
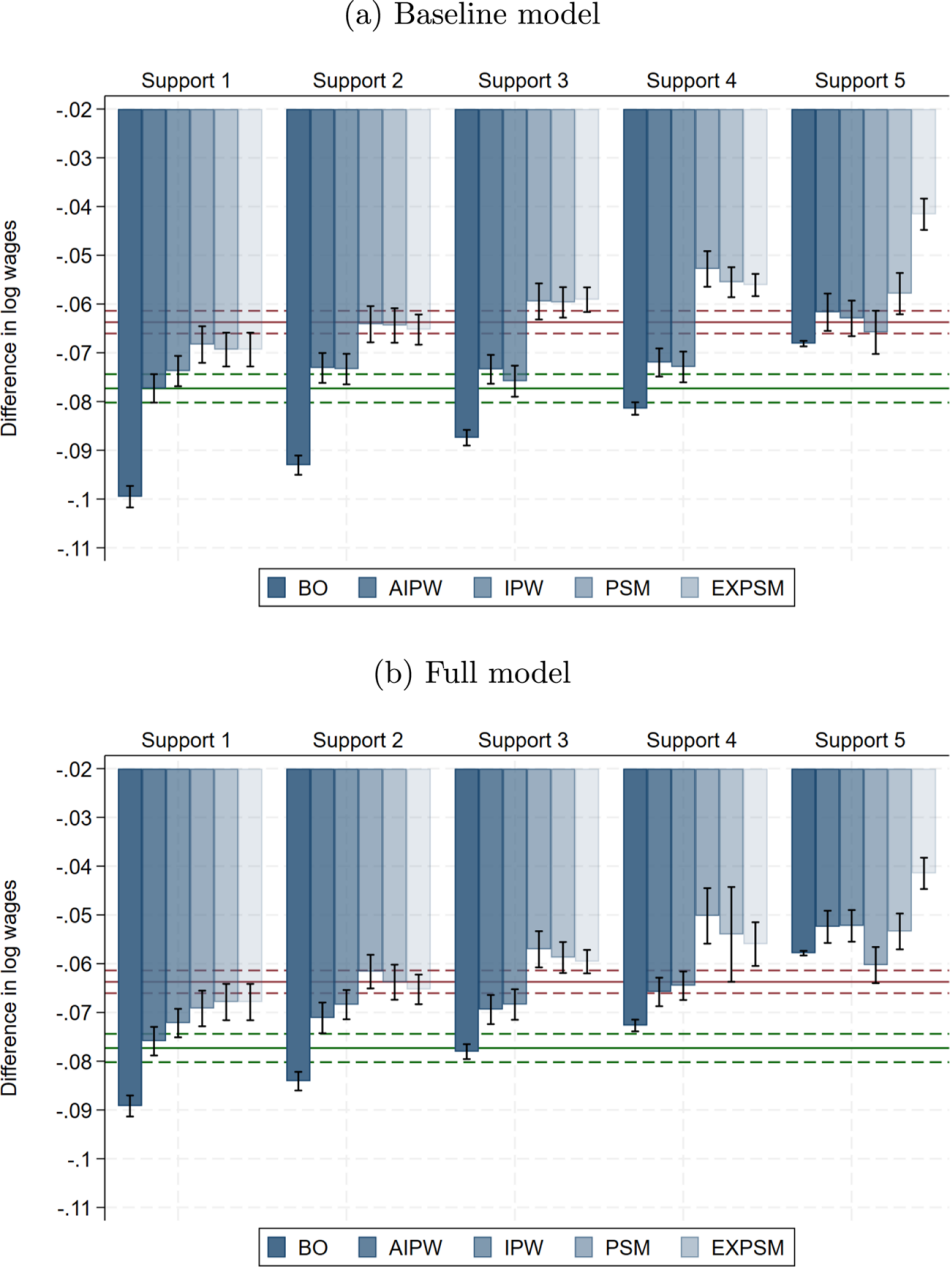
Estimates of the mean unexplained gender pay gap. Notes: The figure displays mean unexplained gender pay gap estimates with 95% confidence intervals (capped lines). The red horizontal line shows the exact matching estimate in Support 3 and its confidence band (red dashed lines); the green line shows the BO estimate in Support 1 and its confidence band (green dashed lines). The results are for the private sector

Figure 3 shows all estimates of the average unexplained gender pay gap we obtain by varying estimators and enforcement of common support with the baseline model specifications for the private sector. The bars show the estimates, and the capped lines show the 95% confidence interval for each estimate. As a non-parametric benchmark, the red horizontal line displays the estimate from exact matching on the variables that define Support 3, which includes all quantitatively important wage determinants, and its 95% confidence band in red dashed lines. It is 2.15 percentage points larger in absolute value than the fully non-parametric estimate that accounts for all observed wage determinants (see Fig. 2). As a second benchmark, the green horizontal line shows the BO estimate in Support 1 with the baseline model specification together with its 95% confidence interval (green dashed lines). This benchmark corresponds to what most studies estimate. We report all estimates, including the full and machine learning model specifications, and their standard errors in Table D.1 in Supplementary material D.^19^

**Fig. 4:**
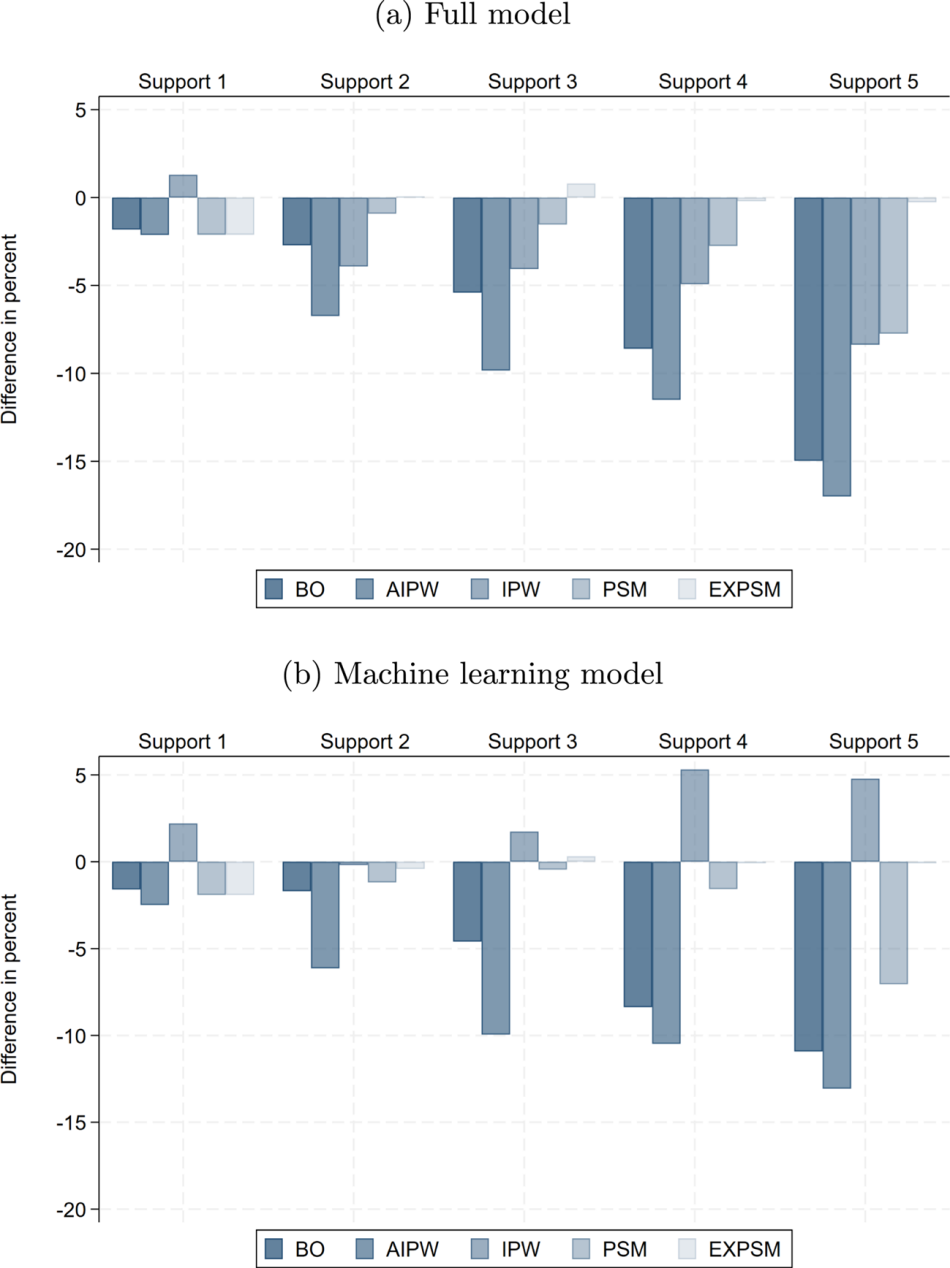
Difference in unexplained gender pay gap relative to baseline model. Notes: This shows the percentage difference in the estimated unexplained gender pay gap relative to the baseline model estimate, by estimator and support version. The results are for the private sector

There are three key insights from Fig. 3. Firstly, all three methodological choices we consider matter greatly. Compared to the BO benchmark, using a less flexible estimator like the LRM increases the estimated unexplained gender pay gap by up to 29%. In contrast, increasing flexibility by including wage determinants more flexibly or by using a more flexible estimator together with enforcing common support reduces the estimated pay gap by up to 53%. These differences are substantial. Secondly, the BO benchmark estimates considerably larger wage gaps than the non-parametric benchmark. This suggests that standard BO estimates may be misleading. Thirdly, the two matching estimators, especially EXPSM, come close to the non-parametric benchmark already with moderate support enforcement (Support 2). In the following, we investigate the role of each methodological choice in detail.^20^ Thereafter, we discuss the joint implications for applied research.

### Role of model flexibility

Figure 4 shows the percentage differences in the estimated unexplained gender pay gaps between the baseline model of the respective estimator and the full and ML model specifications. As expected, model flexibility matters most for the parametric estimator BO, as well as for AIPW, which can be viewed as BO with semi-parametric finite sample bias adjustment. Both model the wage equation to estimate the unexplained gender pay gap, which makes them vulnerable to misspecification. In contrast, the semi-parametric estimators IPW, PSM and EXPSM are much less sensitive to how wage determinants are included. All three estimators only use the propensity score as an input factor and do not impose any functional form on the wage equation.^21^ With the exception of IPW, the differences between the full model and the ML model are very small with less than 5% in most cases.^22^

Among the semi-parametric estimators, EXPSM sticks out. In all but one cases, model specification has a negligible impact on the estimates obtained from EXPSM independent of support. The differences to the baseline model reach at most 2%. This estimator controls for the wage determinants used to enforce common support in a fully non-parametric way. Interestingly, matching exactly on few important wage determinants like in Support 2 seems to suffice to ensure robustness to functional misspecification in the propensity score.^23^

We draw three conclusions from our results. First, it is important to control for wage determinants in a flexible way. Misspecification of functional forms is less likely, and the loss of degrees of freedom is not costly in our very large data set. Second, flexible inclusion of wage determinants is more important for estimators that model the wage equation than for estimators that model only the propensity score. Third, applying ML methods for variable selection from a very rich set of non-linear and interaction terms has only a small impact on the estimated pay gaps. However, this may be different in smaller samples where estimation efficiency is more of a concern. We investigate this further in Sect. 6.5.

### Role of stricter support enforcement

**Fig. 5:**
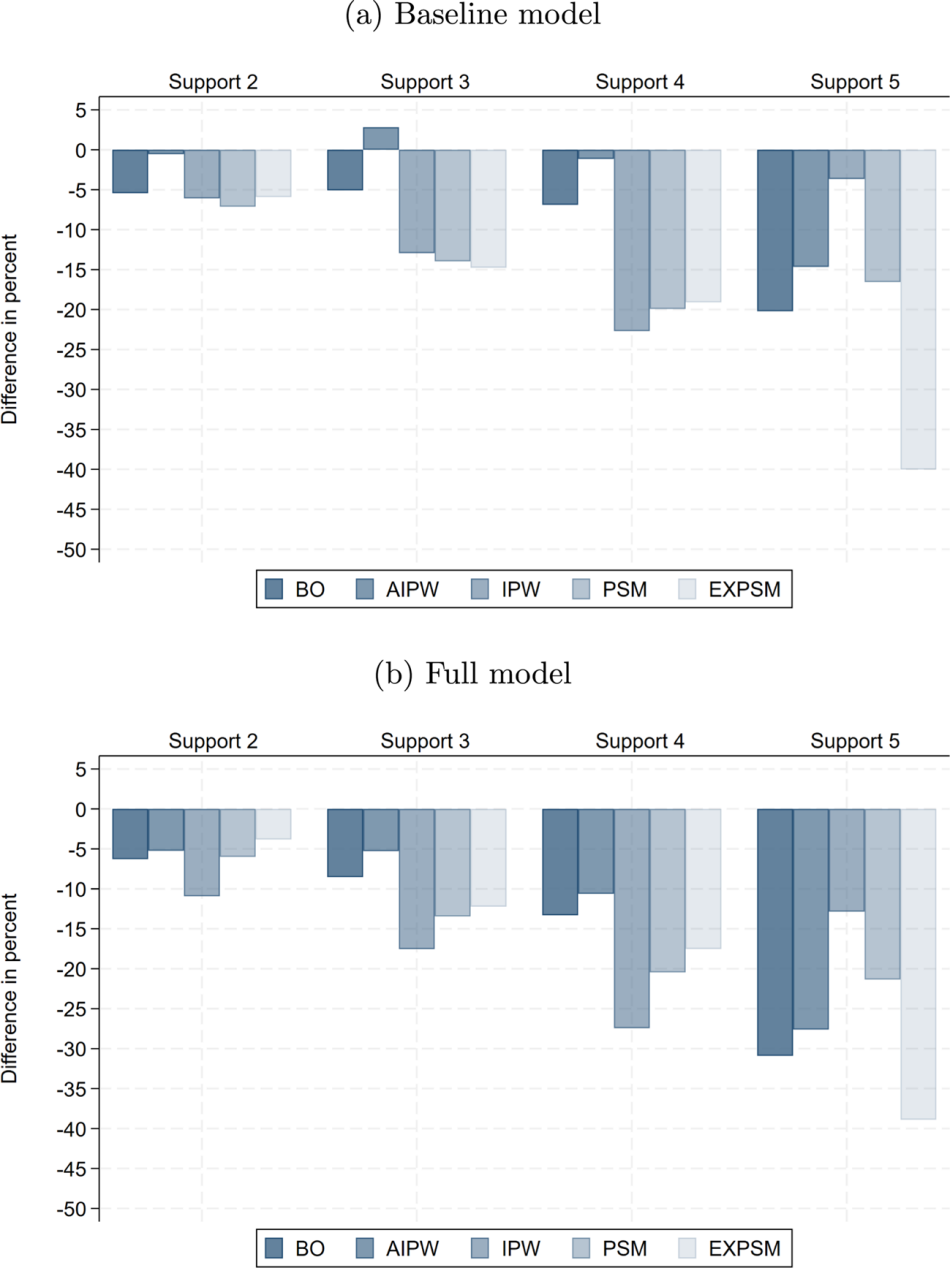
Difference in unexplained gender pay gap relative to Support 1. Notes: This shows, for Support 2 to Support 4, the percentage difference in the estimated unexplained gender pay gap relative to the estimate in Support 1, by estimator. The results are for the private sector

Figure 5 shows the differences in the estimated unexplained gender pay gaps in percent for each support definition relative to Support 1, which includes the full sample. The estimated pay gaps shrink substantially and in most cases monotonically with stricter support enforcement for all estimators and model specifications.

There are two possible explanations for this finding. First, stricter support enforcement makes women and men more comparable, so differences in observed wage determinants are likely to explain a larger share of the raw gender pay gap. Second, heterogeneity in unexplained gender pay gaps may contribute to differences across support samples with increasingly distinct compositions. However, Table 5 shows that compositional differences across support samples are generally modest in our data.

Nevertheless, in principle, stricter support enforcement tends to remove women with part-time jobs. As a result, the unexplained gender pay gap could either increase or decrease, depending on whether unexplained gaps are larger among women in full- or part-time jobs. The direction of this bias cannot be inferred from the data, since the unexplained gender pay gap cannot be estimated for omitted women who lack comparable men.

In the private sector, moderate support enforcement (Support 2) has a relatively small impact on the estimates for all estimators with reductions of close to 5% in most cases. With stricter support enforcement (Supports 3 and 4), the reductions become considerably larger for the semi-parametric estimators (12–28%). They are much smaller for the BO benchmark with only 5–8%. The impact of support enforcement is quite similar across model specifications.^24^

### Role of estimator choice

**Fig. 6:**
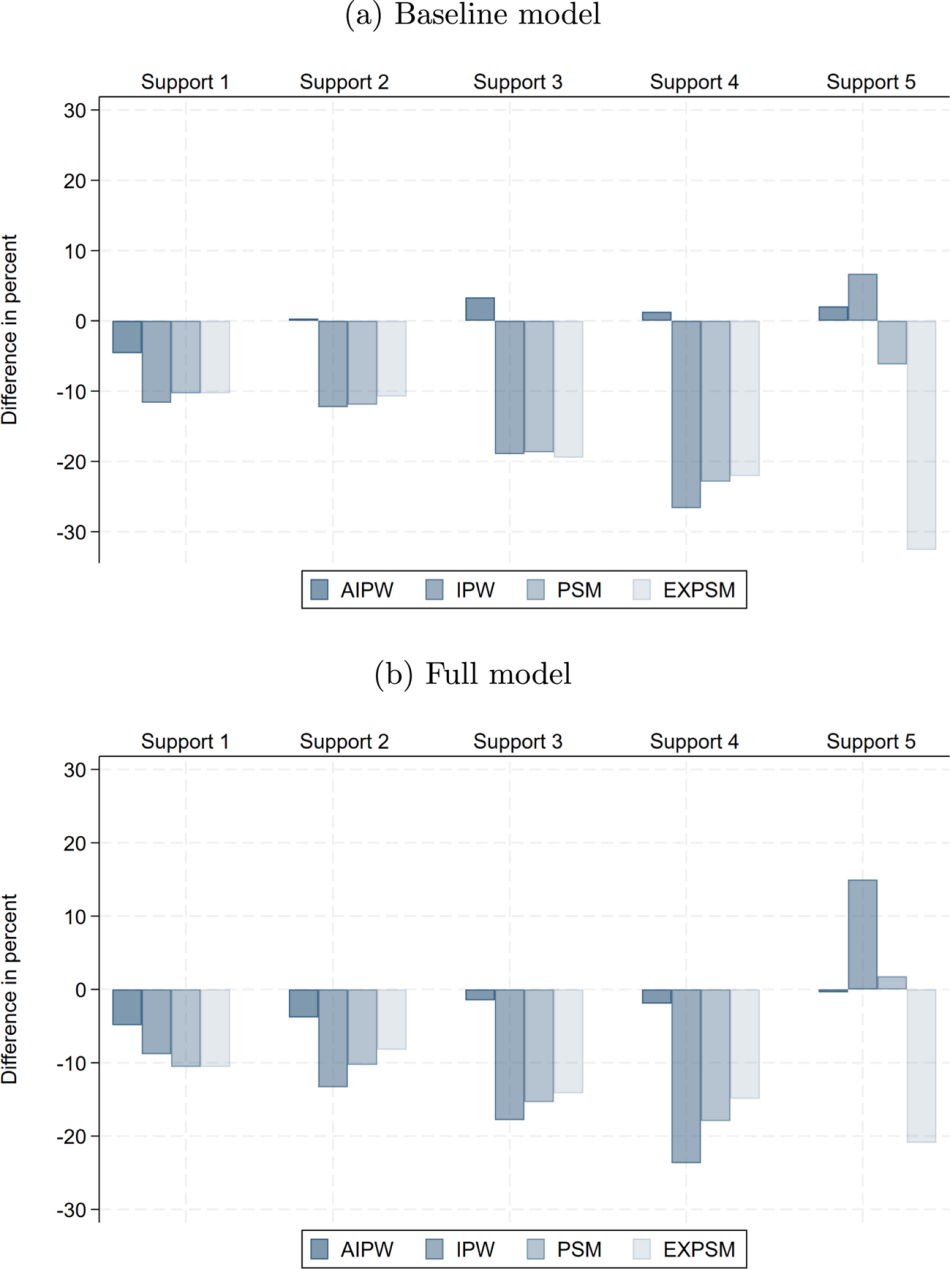
Difference in unexplained gender pay gap relative to BO. Notes: This shows, for different estimators, the percentage difference in the estimated unexplained gender pay gap relative to the BO estimate, by support version. The results are for the private sector

The last dimension we vary is estimator choice. Figure 6 reports the differences in the estimated unexplained gender pay gaps between BO and the respective estimator for each support as well as the baseline and full model specification. We choose BO as the benchmark because it is the work-horse model in applied research.

We find that the AIPW estimates are very similar to BO, as expected with our large samples, but this may be different in smaller samples. With the semi-parametric estimators we obtain pay gaps that are systematically and substantially smaller than the corresponding BO estimates on the same support by up to 33% in the private sector.^25^ The only exceptions are IPW and PSM in Support 5. As expected, more flexible model specifications reduce the differences across estimators in most cases.

Ours are consistent with the results of Frölich (2007b). He documents that the PSM estimates of the unexplained gender pay gap in the UK are up to 29% smaller than the BO estimates. Black et al. (2008) also find strong differences between BO and EXM estimates of the unexplained gender pay gap in the US. In particular, the estimates decline by 18% for white women, 92% for Hispanic women, and 83% for Asian women. However, for black women, they find no strong difference between BO and EXM estimates. Likewise, Ñopo (2008) and Goraus et al. (2017) find no strong differences in BO and EXM estimates of the unexplained gender pay gap in Peru and Poland, respectively, but they only account for a very small set of wage determinants. In Fig. 2, we show that the selection of control variables is crucial for EXM.

Taken together, our findings suggest that the semi-parametric matching estimators PSM and EXPSM exhibit clear advantages over alternative estimators, even when the latter employ reasonably flexible model specifications. Of course, fully saturated models would eliminate all differences across estimators. But they would yield the non-parametric estimate with full support in all wage determinants, which would eliminate all of their advantages. PSM and especially EXPSM come close to the non-parametric estimate, even with moderate support enforcement and less flexible model specifications. They are least sensitive to model misspecification and offer the best solution to the trade-offs between ex ante comparability between men and women, representativeness of the study sample, and precision.

### Role of sample size

The severeness of the curse of dimensionality and the degrees of freedom available to control for wage determinants in a flexible way directly depend on the sample size. In the following, we investigate how common support, estimator choice and model specification affect estimates of the gender pay gap in smaller samples. To this end, we draw 1000 random subsamples of 10,000 observations and 500 subsamples of 100,000 observations, respectively, from the original sample and replicate our analysis within each draw. A sample size of 100,000 is representative of several earlier contributions in the literature (e.g., Black et al. 2008; Meara et al. 2020), whereas 10,000 observations constitute a more constrained empirical setting (e.g., Bonaccolto-Töpfer and Briel 2022; Frölich 2007a) that allows assessing the robustness of methodological choices under more restrictive conditions.

**Fig. 7:**
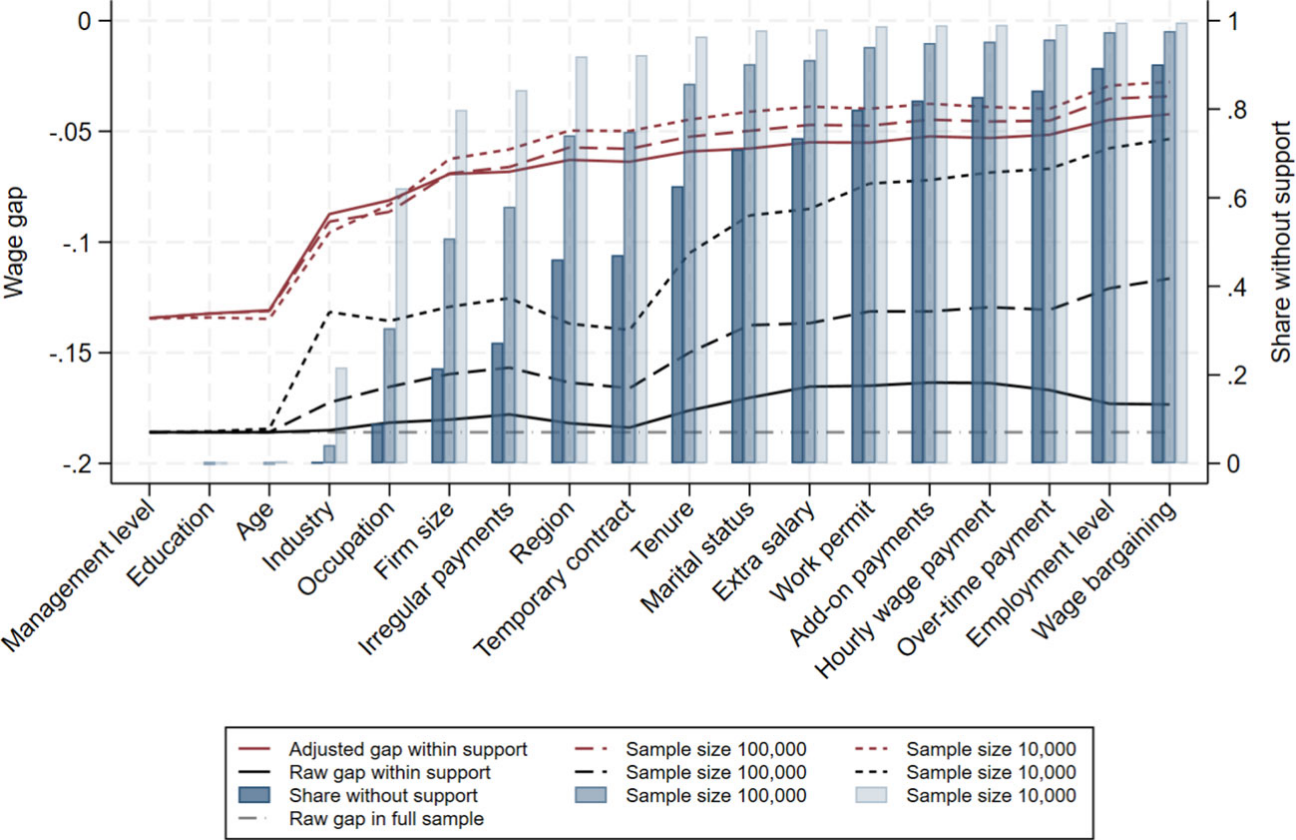
Common support and unexplained pay gaps by sample size. Notes: Wage determinants are added sequentially from left to right. The raw gap in the full sample refers to the difference between the average log wages of women and men without support restrictions imposed. The raw gap within support refers to the difference between the average log wages of women and men in the sample with support, considering all wage determinants from the left up to the respective determinant. The unexplained gap is obtained from exact matching on all wage determinants from the left up to the respective determinant. For sample sizes of 10,000 and 100,000, results are averaged across all random subsamples drawn from the full dataset. The results are for the private sector

Figure 7 shows the results form exact matching when we sequentially add wage determinants for the full sample and the two subsample analyses. For the latter, we report averages over all random draws. For each sample size, the figure shows the share of women without support, the raw wage gap within support and the adjusted wage gap within support obtained from exact matching on the respective variables.

We find that even in much smaller samples, only few women lack support when we match on the three most important wage determinants that define Support 1 in our analysis. Thereafter, however, the share of women without support increases much faster in smaller samples. When matching on all variables in Support 2, this includes all variables up to firm size, on average 27% of women in the samples with 100,000 observations and 84% with 10,000 observation lack comparable men, while this share is only 15% in the full sample. Thus, as expected, common support breaks down much faster in smaller samples.

The raw wage gaps within support become substantially smaller in smaller samples, implying that the share of the total raw gap attributable to lack of support is considerably larger than in the full sample. Specifically, it reaches up to 37% with 100,000 observations and 71% with 10,000. This pattern is plausible, as the remaining sample becomes more homogeneous more quickly when support deteriorates faster. Interestingly, the unexplained wage gaps obtained from exact matching are very similar across samples of different sizes when restricting to the five most important wage determinants. When adding more variables, the unexplained gaps become even smaller than in the full sample, reaching their lowest values with a sample size of 10,000. Thus, the non-parametric benchmark we obtain in the full sample represents a lower bound relative to smaller samples.

**Fig. 8:**
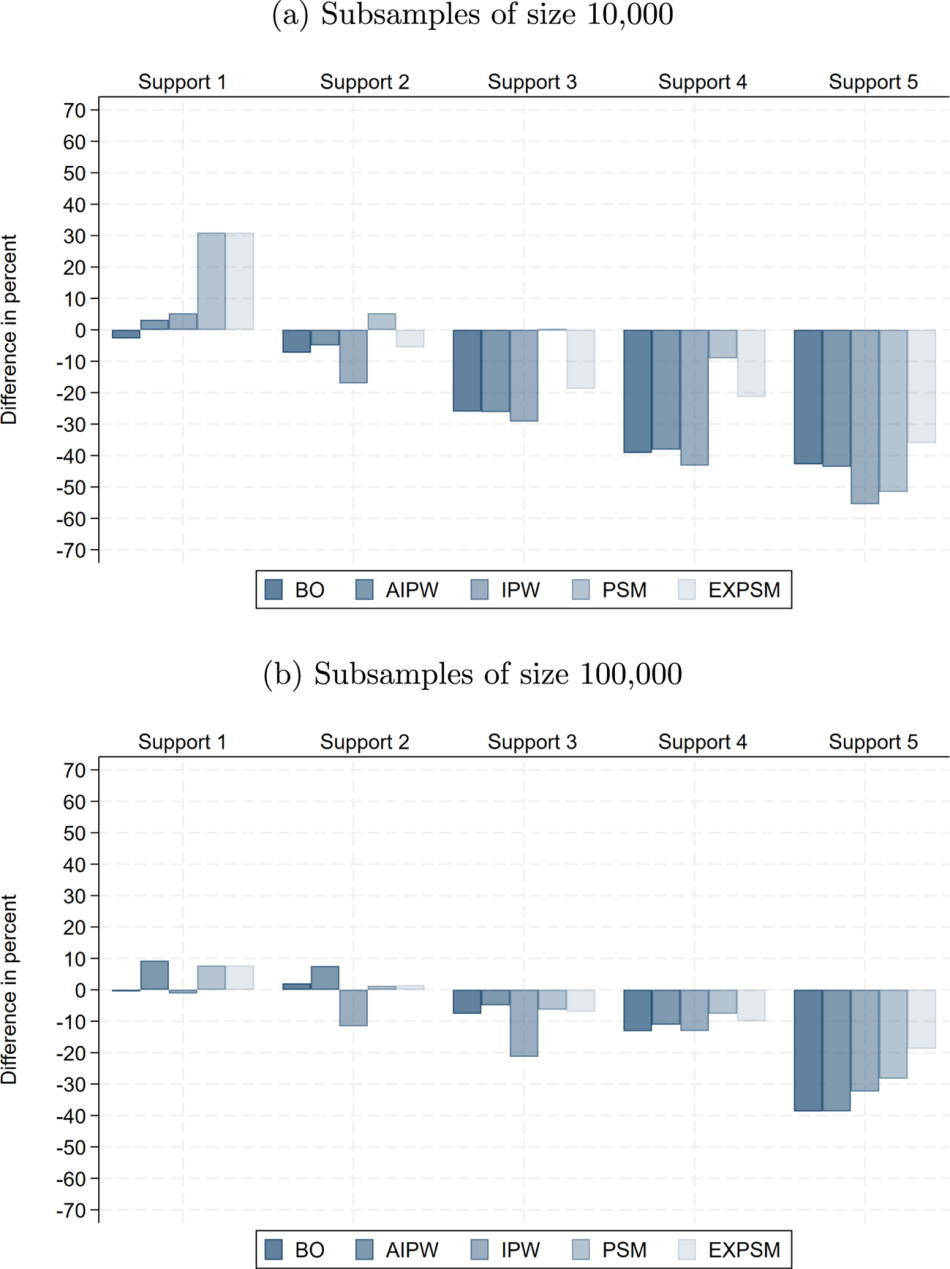
Difference in adjusted gender pay gap relative to full sample: baseline model. Notes: This shows the percentage difference between the average estimated unexplained gender pay gap in the subsamples and the corresponding estimate in the full sample, for the same estimator, support version, and model specification. The results are for the private sector

**Fig. 9:**
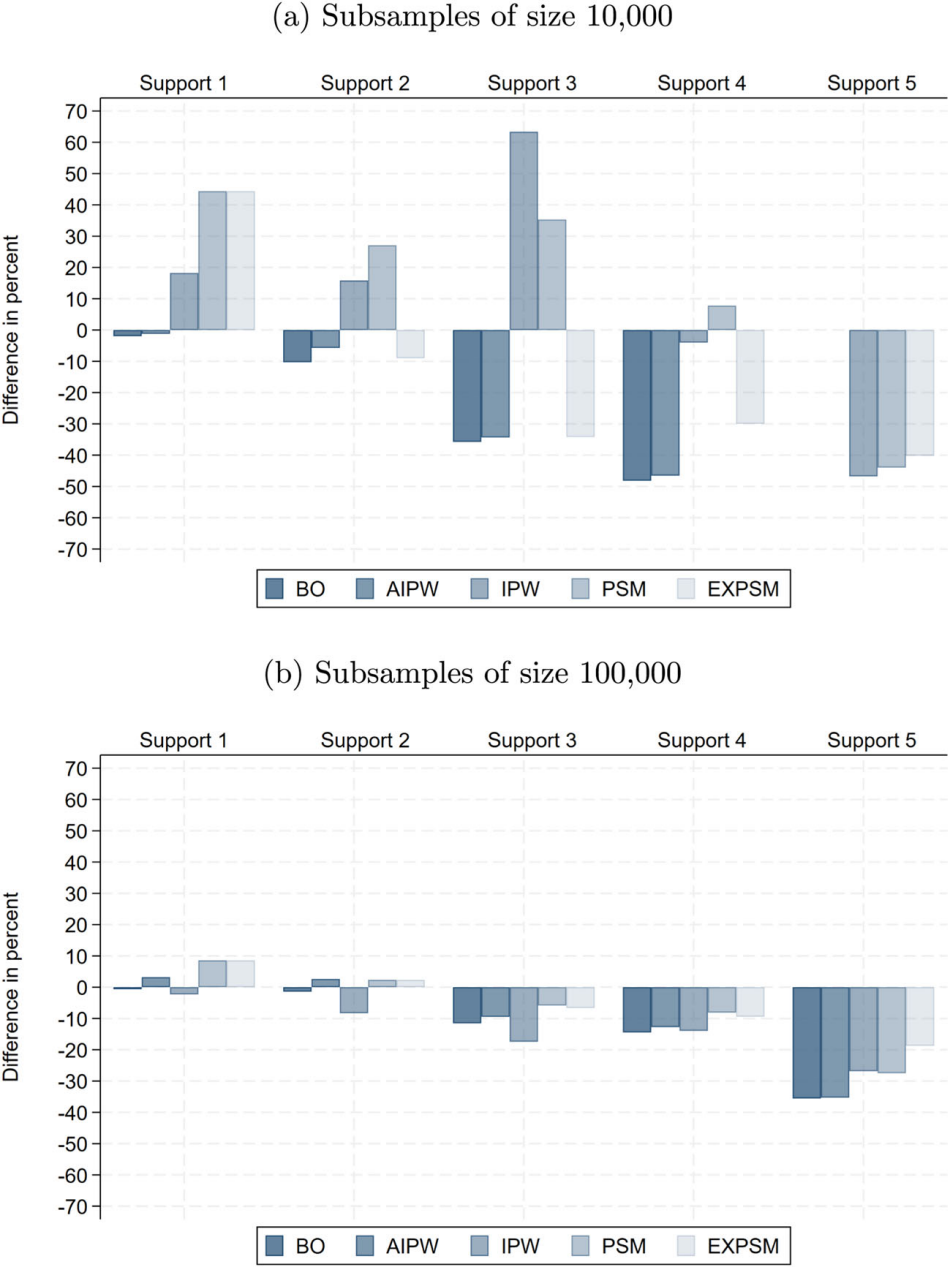
Difference in adjusted gender pay gap relative to full sample: full model. Notes: This shows the percentage difference between the average estimated unexplained gender pay gap in the subsamples and the corresponding estimate in the full sample, for the same estimator, support version, and model specification. Results for BO and AIPW in Support 5 in subsamples with 10,000 observations are omitted because they fail to produce meaningful estimates. The results are for the private sector

**Fig. 10:**
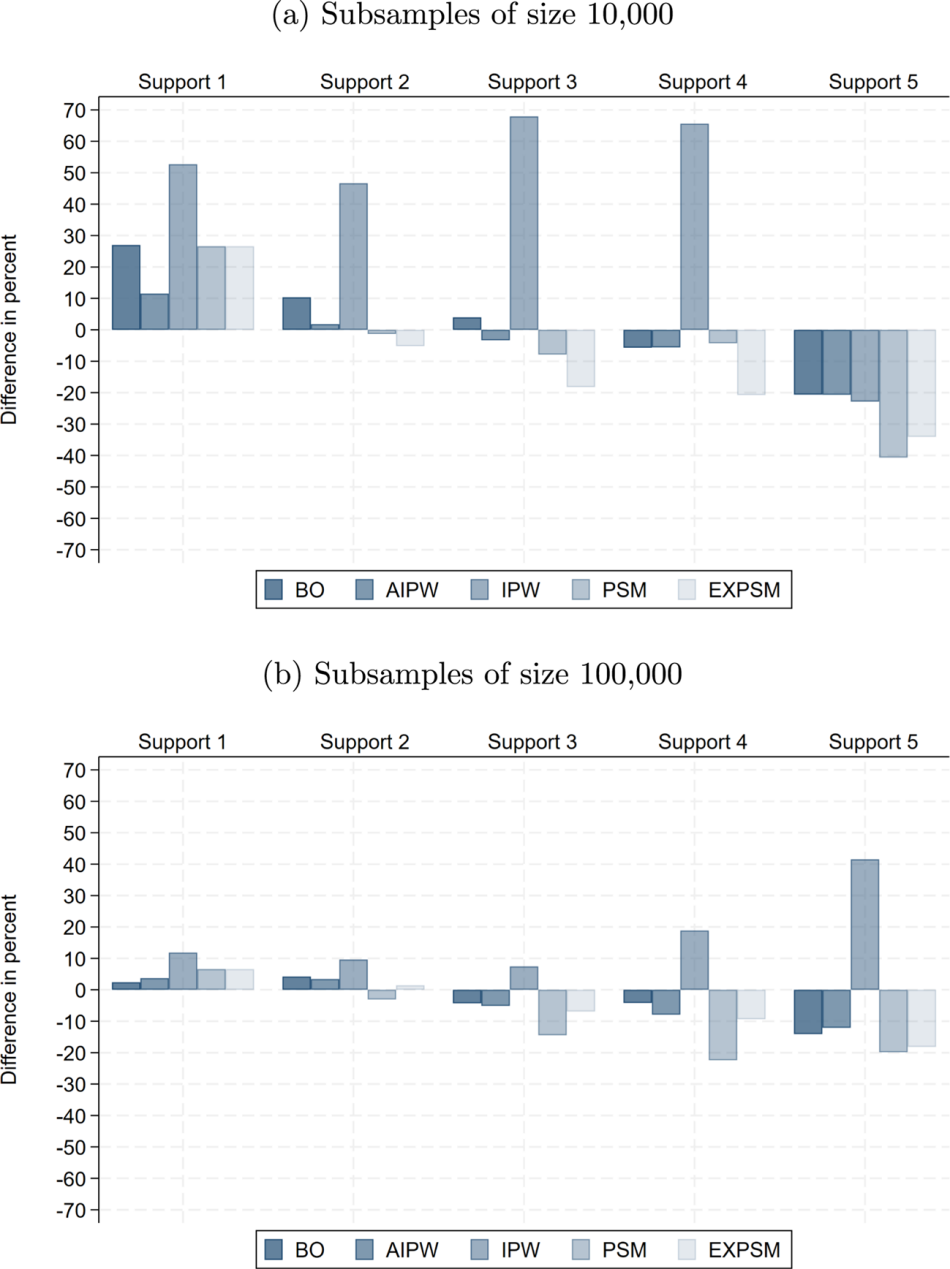
Difference in adjusted gender pay gap relative to full sample: ML model. Notes: This shows the percentage difference between the average estimated unexplained gender pay gap in the subsamples and the corresponding estimate in the full sample, for the same estimator, support version, and model specification. The results are for the private sector

Before we discuss the results for the unexplained wage gap obtained from alternative estimators, it is important to examine how the sample size and composition changes when stricter support enforcement is applied in smaller samples. Tables D.6 and D.5 in Supplementary material D report the means of the wage determinants across Supports 1–5, as defined in Table 4, the percentage differences relative to the full sample within each support, and the average size of the remaining sample. As expected under random sampling and almost full overlap in Support 1, the compositional differences to the full sample are very small in Support 1 for both sample sizes. With stricter enforcement, however, sample composition diverges increasingly and most rapidly in the smallest samples, reflecting the accelerated breakdown of support.

Interestingly, the differences between Supports 2 and 5 in the samples with 10,000 observations and between Supports 3 and 5 in the samples with 100,000 observations are relatively small. This pattern coincides with the points where lack of support is already very high and increasing further only slowly. Another important insight from Table D.6 is that Supports 4 and 5 in subsample 10,000 contain, on average, only 331 and 35 women, respectively. These are fewer than the 615 variables included in the full model specification. Moreover, in Support 3, the number of observations drops to 722, leaving very few degrees of freedom. Therefore, overfitting becomes a significant concern in these settings, and machine learning approaches can help mitigate this issue.

Figures 8, 9, and 10 show how the estimates of the unexplained gender pay gap in the 10,000- and 100,000-observation subsamples differ from those in the full sample. For the 100,000-observation subsamples, a clear and consistent pattern emerges, with the exception of IPW. First, except under Support 5, differences relative to the full sample remain modest and typically below 10%. Second, as support enforcement becomes stricter, deviations from the full sample benchmark shift systematically from positive to negative. That is, for moderate levels of support enforcement, estimated gaps in the subsamples are up to 10% higher than in the full sample, while under stricter support versions they are up to 10% lower. Third, for most estimators, estimates based on the ML model are closer to the full sample benchmark than those obtained from the other two model specifications, underscoring the value of ML for model specification in 100,000-observation samples.

In the more constrained setting with 10,000 observations, deviations from the full sample are naturally larger, especially under Supports 3 to 5. In the baseline model, we again observe a systematic shift from positive to negative deviations as support enforcement becomes more restrictive. By contrast, the full model shows the largest discrepancies and fails to deliver meaningful estimates for BO and IPW under Support 5, where the number of available observations falls below the number of covariates (these estimates are omitted from the figure). Beyond Support 1, ML estimators perform considerably more robustly, producing estimates that remain relatively close to those in the full sample. The notable exception is IPW, which again displays instability even when combined with ML.

Taken together, our results for different sample sizes provide three key insights. Firstly, as expected, only moderate enforcement of common support is feasible in small samples. Even Support 2 may be too restrictive in practice. A pragmatic alternative is to redefine categorical variables using broader groupings, such as aggregating industry or occupation codes to the one-digit level, thereby preserving overlap while maintaining meaningful comparability. Secondly, employing machine learning for model specification offers a valuable strategy to navigate the trade-offs between model flexibility and efficiency in smaller samples. However, this advantage diminishes as sample size increases, where the full specification can be employed without compromising estimation efficiency. Thirdly, under moderate definitions of support, estimates of the unexplained gender pay gap remain highly stable across different sample sizes. This stability implies that the key conclusions regarding estimator choice are not confined to large-scale data environments, but hold more broadly across empirical settings with considerably fewer observations.

### Implications for applied research

Our results have important implications for applied research. The first key insight is that studying gender-based sorting beyond comparing mean characteristics is important, even when using parametric estimators. We recommend starting with an analysis of common support and the resulting raw and unexplained gender pay gaps using the exact matching approach of Ñopo (2008). Firstly, this exercise informs about the trade-offs in terms of sample size and to which extent ex ante enforcement of common support is feasible. Secondly, it reveals which part of gender inequality in pay is due to lack of support. Thirdly, it offers a benchmark to which chosen estimators can be compared.

The first important decision is whether and how to enforce common support ex ante. In a given application, this decision depends on the sample size, the extent of lack of support and how much it explains gender inequality in pay. Small samples and strong sorting limit the possibility to enforce support ex ante. At the same time, support enforcement is more important when lack of support explains a larger part of the raw gender pay gap. Our findings suggest that it is sufficient to enforce support with respect to few very important wage determinants, especially when combined with a flexible estimator. This approach offers a good trade-offs between ensuring comparability of men and women ex ante, and precision and representativeness of estimated covariate-adjusted gender gaps. If lack of support is substantial, we advise to make transparent how different support definitions affect sample composition and estimation results.

The second important decision is which estimator to use. Our results indicate that restricting the wage equation can have a large impact on the estimate. When using BO, including wage determinants in a flexible way is crucial. However, with a sufficiently large sample, a matching estimator with flexible specification of the propensity score is preferable, as matching does not restrict the wage equation in any way. In both cases, machine learning helps to find the optimal trade-offs between model flexibility and efficiency in specifying either the wage equation or the propensity score, especially in smaller samples. Among the semi-parametric estimators, we find that PSM and EXPSM are the most robust and precise. Our findings suggest that combining exact matching on few very important wage determinants that also define support with radius matching works particularly well. It minimizes the risk of functional form misspecification and offers a good balance between ensuring comparability, precision, and representativeness of the study sample.

Table 6 shows how implementing these recommendations affect the results in our application. Support 2 corresponds to very moderate support enforcement, while Support 3 would be more conservative. The standard approach would be to use BO without support enforcement (Support 1) and the baseline specification. This results in an unexplained wage gap of 7.7% and explains 58.6% of the raw wage gap in the private sector. Enforcing common support reduces the unexplained gap by no more than 5%. Using the most flexible specification with BO reduces the estimated unexplained wage gap considerably by 8–10% with larger reductions when support enforcement is more strict. Using EXPSM instead of BO strongly affects results for all support definitions and model specifications. The adjusted wage gap is 10–23% smaller compared to standard BO in the private sector. Model specification has a negligibly effect on the EXPSM estimates, suggesting that the baseline specification would suffice. Estimates become smaller with stricter support enforcement, but the differences between Supports 2 and 3 are moderate.

**Table 6:** Unexplained gender pay gaps, share explained, and deviations from standard BO estimates

Support	Model	Unexplained	Share	Difference to
version	specification	gap	explained	standard BO
		Blinder-Oaxaca (BO)		
1	Base	$-$7.7	58.6	–
2	Base	$-$7.3	60.7	$-$5.2
3	Base	$-$7.3	60.7	$-$5.2
1	Full	$-$7.6	59.1	$-$1.3
2	Full	$-$7.1	61.8	$-$7.8
3	Full	$-$6.9	62.9	$-$10.4
		Combined exact and PSM (EXPSM)		
1	Base	$-$6.9	62.9	$-$10.4
2	Base	$-$6.5	65.0	$-$15.6
3	Base	$-$5.9	68.2	$-$23.4
1	Full	$-$6.8	63.4	$-$11.7
2	Full	$-$6.5	65.0	$-$15.6
3	Full	$-$6.0	67.7	$-$22.1

Notes: This reports, for BO and EXPSM, the estimated unexplained gap (in percent), the share of the raw wage gap explained (in percent), and the difference between the respective unexplained gap estimates and standard BO in Support 1 with the baseline specification (in percent). The raw gender pay gap in the private sector is $-$18.6%

In our application, we recommend using EXPSM in Support 2 with the baseline specification and implementing Support 3 as robustness check. Support 2 leads to moderate loss of observations and keeps the representativeness of the remaining sample largely intact. In the private sector, this results in an unexplained wage gap of 6.5%. This is 15.6% lower than the standard BO estimate and explains 11% more of the raw wage gap. With Support 3, we obtain a gap of 6% that is 23.4% lower than standard BO and accounts for 16% more of the raw gap.^26^ This illustrates how important these methodological choices are.

## Conclusion

This study examines to which extent gender sorting in the labor market results in lack of comparability between men and women and how this affects estimates of covariate-adjusted gender pay gaps. We document that sorting is substantial in the Swiss labor market and results in a sizeable share of workers without employees of the opposite gender that are comparable in important wage determinants. We show that this limits the usefulness of the exact matching approach of Ñopo (2008) as the non-parametric ideal when many wage determinants are relevant, even in datasets with over a million observations.

Exploring alternative estimation choices, we find that ex ante restrictions of support, estimator choice, and model specification for a given estimator matter greatly when estimating covariate-adjusted gender pay gaps. Enforcement of common support ex ante strongly affects estimates, especially when lack of support explains a large share of the raw gender pay gap. For estimators that model the wage equation like the LRM or BO, it is important to include wage determinants in a flexible way to reduce the risk of extrapolating into regions without support based on misspecified functional forms. Using semi-parametric PSM, which does not impose any restrictions on the wage equation, lowers this risk further. Our results further suggest that using a more flexible estimator allows for imposing fewer support restrictions, which has advantages in terms of precision and representativeness of the estimates. We find that combining exact matching on few important wage determinants with PSM performs particularly well. It minimizes the risk of functional form misspecification and is able to capture gender inequality in pay due to lack of support. Thus, it offers an attractive alternative to exact matching when the latter suffers from the curse of dimensionality caused by a large number of relevant wage determinants, as is common in modern applied work.

Our results show that BO estimates of the unexplained gender pay gap, which are most common in applied work, can be misleading as the restrictions they impose can yield biased estimates. There is a high risk that they compare women with non-comparable men to an extent that is quantitatively very important. Moreover, they hide gender inequality in pay that results from this lack of comparability. The reliability of inputs for policy makers could be improved considerably by applied researchers paying more attention to lack of comparability between men and women and estimation choices. Ideally, assessing lack of support and how it affects the measurement of wage inequality would become a standard procedure when studying inequality in pay. For policy makers, this also has direct value because it informs about the extent of sorting in the labor market and can uncover a potentially important source of wage inequality that has received little attention in applied work so far.

## Supplementary Information

Below is the link to the electronic supplementary material.

Supplementary file 1 (pdf 357 KB)

## Data Availability

The paper uses the Swiss Earnings Structure Survey 2016 under contract number 240607. The contract prevents us from making the data publicly available. However, there is a standardized procedure for obtaining access to the data for research purposes from the Swiss Federal Statistical Office via lohn@bfs.admin.ch. We are happy to provide all necessary information about the data application process, and we are willing to share our codes and programs for the data preparation and estimation.

## Footnotes

1 Bundesamt für Statistik, Schweizerische Lohnstrukturerhebung (LSE) 2016.

2 All results we report take into account the sampling weights provided by the ESS that correct for both stratification, and non-response.

3 See Bayard et al. (2003); Beaudry and Lewis (2014); Bertrand et al. (2019); Bruns (2019); Buffington et al. (2016); Card et al. (2016); Goldin et al. (2017) and Sin et al. (2022) among others.

4 See, e.g., Flory et al. (2015) and Gneezy et al. (2003) for competitiveness; Angelov et al. (2016); Bailey et al. (2012); Kleven et al. (2019) and Lundborg et al. (2017) for children; Bertrand et al. (2015) for gender norms; and Fortin (2008) for non-cognitive factors.

5 We investigate the quantitatively important wage determinants in Sect. 4.1. The SES contains all wage determinants we use to define Supports 1–4 (but not 5).

6 Based on two-digit ISCO-08 codes 01, 02, 03, 61, and 62.

7 Based on two-digit NOGA 2008 codes 01, 02, 05, and 12.

8 Based on two-digit NOGA 2008 codes 10, 31, 45, 47, 55, and 58.

9 There are some notable differences across sectors. With 56% of employees, women are over-represented in the public sector. Only 43% of workers in the private sector are females. Public-sector jobs are concentrated in knowledge-intensive services like education, health, and public administration. Accordingly, more employees hold a university degree and occupations represented in the public sector are more homogeneous. Tenure is also considerably longer than in the private sector speaking to less turnover and more job security. In terms of gender differences, the patterns are similar to the private sector, though. The differences we find between the private and the public sectors in Switzerland are present in many other countries. Public sectors in most countries share the features that explain the differences in the Swiss case. They are typically more homogeneous in terms of covered occupations with a strong concentration in certain service sectors, they typically attract a higher share of women than the private sector, they exhibit higher shares of high-skilled workers, they offer more secure jobs, and they are typically more regulated, including their wage setting, than private sectors (see Brindusa et al. 2012).

10 Similarly, by adding and subtracting $E_{X|G=0}[\mu _1(x)]$, one can decompose the raw gender pay gap into the unexplained gap for males and the explained gap that equates the covariate distributions across gender using the wage of females as benchmark.

11 Similarly, if interest is in the unexplained pay gap for males, the empirical counterpart of $E_{X|G=0}[\mu _1(x)]$ only exists if $0<Pr(G_i=1|X_i=x)\le 1$. The condition $0<Pr(G_i=1|X_i=x)<1$ ensures common support for both males and females.

12 We report the corresponding results for the public sector in Table B.2 in Supplementary material B. The order of variables affects the evolution of common support and the estimated unexplained wage gaps as we add variables but not their value for a given combination of variables. In particular, it has no effect on what we obtain with enforcement of full support with respect to all observed wage determinants. We show the results for alternative orders in Table B.3 in Supplementary material B.

13 In the public sector, common support deteriorates more slowly. When enforcing support with respect to the ten variables most important for explaining male wages, 32% of women in the public sector have no comparable men. When enforcing full support across all observed wage determinants, 70% of women in the public sector lack a comparable male counterpart. The slower deterioration in the public sector reflects the fact that it is more homogeneous in terms of occupations and industries compared to the private sector.

14 This is very different in the public sector. Here, up to 68% of the raw gap is due to lack of support (see Table B.2 in Supplementary material B).

15 The decrease is much stronger for the public sector, where the gaps fall quickly from 9.7 to 3.5% when we impose support with respect to the eight most important wage determinants.

16 Bach et al. (2024) and Imai and Ratkovic (2013) propose machine learning approaches to obtain unbiased estimates of $\alpha _{BO}$ and $\beta _{BO}$. We focus on Eq. 5.8 to implement BO because we are mainly interested in $\delta _{BO}$ and not in the structural parameters $\alpha _{BO}$ and $\beta _{BO}$.

17 It should be noted that we implement PSM after matching exactly on the three variables that define the least restrictive Support 1 (see Sect. 5.4) because this improves matching without lack of support. As a result, PSM and EXPSM are identical for Support 1 but not for the other support definitions.

18 Table B.6 in Supplementary material B documents the corresponding results for the public sector.

19 Correspondingly, we report all results for the public sector in Table D.2 in Supplementary material D.

20 As the LRM is clearly inferior to all other estimators in all cases we consider, we do not discuss it further in the main text.

21 Kline (2011) provides a theoretical justification for the sensitivity of the BO estimator with regard to model flexibility. He argues that BO is more vulnerable to model misspecification than IPW because the implicit weighting scheme of BO allows for negative weights (which is not permitted for IPW). When the BO model is specified more flexibly, though, negative implicit weights become less likely.

22 Knaus et al. (2021) document low finite sample performance of IPW in combination with ML, which may explain this result.

23 There are interesting differences across sectors. Except for Support 5 as the most extreme case, model specification has a much stronger effect on the estimates in the public sector than in the private sector. The differences in the public sector are up to 20% for the parametric estimators but no more than 10% in the private sector. For the semi-parametric estimators, they reach up to 12% in the public sector versus 5% in the private sector. The key difference between sectors is that a relatively large part of the raw gender pay gap in the public sector is due to lack of common support, while this is not the case in the private sector. Our results suggest that model misspecification is more problematic when lack of common support explains a larger part of observed wage inequality.

24 In the public sector, imposing stricter support requirements has a systematically larger impact on the semi-parametric estimates than the parametric ones. With 22–40% compared to Support 1, the difference is particularly large for EXPSM. By matching exactly on the variables that define support, EXPSM is the only estimator that fully captures the difference in the raw wage gap due to lack of support.

25 In the public sector, the semi-parametric estimators are up to 48% smaller than the corresponding BO estimates on the same support.

26 Differences are much larger in the public sector. Here, Support 2 yields an adjusted wage gap of 3.6%. Compared to standard BO, this is 43.8% smaller and accounts for 38% more of the raw gap. Support 3 reduces the unexplained gap to 3.2%, which is only half of the standard BO estimate, and explains 43% more of the raw gap.
