# Supplementary material for: Labor market sorting and the gender pay gap revisited
Source: J Popul Econ. 2025 Jul 8;38(3):59. doi: 10.1007/s00148-025-01115-1 (PMC12238104; doi:10.1007/s00148-025-01115-1)
Supplement: Supplementary file 1 — (pdf 357 KB) [file 148_2025_1115_MOESM1_ESM.pdf]

Online Appendix
Labor Market Sorting and the Gender Pay Gap Revisited

Anthony Strittmatter* Conny Wunsch†

June 8, 2025

Contents

A Variable Description **2**

B Common Support **4**

C Machine learning model (ML) **11**

C.1 Differences across estimators 11

C.1.1 Post-double-selection (PDS) procedure for the LRM 12

C.1.2 T-learner for BO 13

C.1.3 Cross-fitting for AIPW 13

C.2 Selected variables 14

C.3 Performance. 14

D Full Estimation Results **18**

*UniDistance Suisse, Switzerland; further affiliations with CREST-ENSAE, University of Johannesburg and CESifo; email: anthony.strittmatter@unidistance.ch.

†Faculty of Business and Economics, University of Basel, Switzerland; further affiliations with CESifo, DIW, IZA; email: conny.wunsch@unibas.ch.

A Variable Description

Table A.1: List and Description of all Variables we Use for Exact Matching

Variable	Description	Values
Standardized monthly wage	Full-time equivalent monthly gross wage incl. add-ons, 1/12 of irregular payments and 1/12 of extra salaries	
Occupation	ISCO-08 classification (2 digits)	Private sector: 40 groups, public sector: 24 groups
Management level	Management level	None, lower, middle, upper, top management
Education	Highest level of education	No vocational degree, lower/middle/higher vocational degree, University or equivalent
Age	Age in years	20-29, 30-39, 40-49, 50-59
Tessin	Dummy for Tessin region	Tessin yes/no
Firm size	Number of employees in establishment	< 20, $20 - 49$, $50 - 249$, $250 - 999$, > 999
Irregular payments	Has received bonus or other irregular payments	Yes/no
Tenure	Tenure in years	0-1, 2-4, 5-7, 8-15, 16-45
Region	Swiss greater regions	Geneva, Espace Middleland, Northwestern Switzerland, Zurich, East Switzerland, central Switzerland, Tessin
Industry	NOGA 2008 classification (2 digits)	Private sector: 36 groups, public sector: 12 groups
Temporary contract	Temporary contract	Yes/no
Wage bargaining	Company has collective wage agreement	Yes/no
Extra salary	Extra 13th or 14th salary	Yes/no
Add-on payments	Extra remuneration for night, shift or Sunday work or other non-standard working conditions	Yes/no
Over-time payment	Extra remuneration for over-time work	Yes/no
Hourly wage payment	Wage calculation based on hourly wage	Yes/no
Marital status	Marital status	Single, married, other
Employment level	Percent of full-time job	20-49%, 50-79%, 80-99%, 100%
Work permit	Nationality and work permit for foreigners	No permit: Swiss, short-term permit (b), long-term permit (c), other

Table A.2: Means and Standardised Differences: Public Sector

	Mean		Std.
	Women	Men	Diff.
	(4)	(5)	(6)
Wage			
Standardised monthly wage (in CHF)	7,731	8,985	42.2
Demographics			
Age	41.78	43.28	14.5
Education			
University	.32	.35	5.9
Vocational	.50	.51	2.5
No vocational	.07	.05	11.0
Job Characteristics			
Part-time	.71	.27	98.6
Tenure	8.29	9.56	15.2
Management level			
Top	.01	.04	18.7
Upper	.08	.11	11.6
Middle	.07	.11	14.8
Lower	.06	.08	8.2
None	.74	.61	26.2
Irregular wage components (e.g., bonuses)	.14	.23	23.3
Occupation (ISCO 1-digit)			
Managers	.05	.10	20.2
Professionals	.41	.38	5.1
Technicians & Associate Professionals	.30	.24	12.3
Clerical Support Workers	.08	.03	20.3
Services & Sales Workers	.12	.14	5.5
Craft & Related Trades Workers	.01	.04	18.8
Plant & Machine Operators & Assemblers	0	0	0
Elementary Occupations	0	0	0
Employer Characteristics			
Industry			
Less knowledge-intensive services	.01	.03	20.1
Knowledge-intensive services	.99	.91	33.9
Other (incl. construction)	.01	.06	26.5
Firm size			
< 20	.02	.02	1.4
20–49	.03	.03	1.1
50–249	.12	.09	10.3
250–999	.12	.11	3.0
≥ 1000	.72	.76	9.4
Observations	227,617	177,831	

Notes: The table presents mean values by gender and the standardised differences (std. diff.) between women and men, based on the baseline sample prior to imposing any support restrictions. Monthly regular wages are standardised to 100% full-time equivalents and exclude overtime hours.

B Common Support

Tables B.1 and B.2 show the full results for implementing the approach of Ñopo (2008) in our data when we add variables in decreasing order of importance for predicting wages. Different orders of variables lead to different evolutions of support. Table B.3 shows how support changes when we add these variables in three alternative orders. The first one uses *sector-specific* R^2 changes rather than the average over both sectors to order variables. For the main analysis, we want to keep the results comparable across sectors, which is why we want the same order for both sectors. The order resulting from *sector-specific* R^2 changes differs across sectors for some variables, but the overall differences are moderate. Accordingly, the evolution of support when adding variables is also similar. The second alternative is a random order. Here support remains higher for a larger number of added variables, mainly because critical variables such as industry and tenure are, by chance, added relatively late. This would change with a different random order in which the are added earlier. The last alternative uses an *increasing* order according to the sector-average R^2 changes as the other extreme to a decreasing order. Here, support breaks down rather late. The reasons for this are twofold. First, the ordering prioritises wage determinants in which women and men do not greatly differ. Second, many of these wage determinants are dummies while more important variables (such as education, occupation and industry) have many different categories. This mechanically splits the sample in more cells than a dummy variable such that cell size is reduced.

Tables B.4 to B.6 provide complementary descriptive statistics for our analysis of common support. Table B.4 presents the results for the public sector and is the direct analogue to Table 5, which reports the corresponding results for the private sector. Tables B.5 and B.6 document the number of observations retained under different support definitions, trimming procedures, and matching approaches, separately for the private and public sectors.

Table B.1: Common Support and Results from Exact Matching in the Private Sector

Variable added	Change in Adj. R^2 in %	Without support $S_i = 0$ Share Total	Women	Men	Raw gap Δ	SE	With support $S_i = 1$ Raw gap Δ	SE	Unexplained δ	SE	Explained η	SE	Difference to total raw gap ρ	SE
Management position	7.92	0	0	0	-	-	-0.186	0.001	-0.134	0.001	-0.051	0.001	0	0
Education	6.80	0	0	0	-	-	-0.186	0.001	-0.132	0.001	-0.054	0.001	0	0
Age (1)	3.93	0	0	0	-	-	-0.186	0.001	-0.131	0.001	-0.055	0.001	0	0
Industry	5.80	0.003	0.001	0.005	-0.394	0.038	-0.185	0.001	-0.087	0.001	-0.098	0.001	-0.001	0.000
Occupation	2.60	0.090	0.049	0.121	-0.191	0.005	-0.181	0.001	-0.081	0.001	-0.100	0.002	-0.004	0.000
Establishment size (2)	1.93	0.215	0.145	0.268	-0.182	0.003	-0.180	0.001	-0.069	0.002	-0.111	0.002	-0.006	0.001
Irregular payments	2.01	0.273	0.194	0.332	-0.178	0.003	-0.178	0.001	-0.068	0.002	-0.110	0.002	-0.008	0.001
Region	1.08	0.461	0.375	0.526	-0.178	0.002	-0.182	0.002	-0.063	0.001	-0.119	0.002	-0.004	0.001
Temporary contract (3)	0.01	0.471	0.386	0.534	-0.178	0.002	-0.184	0.002	-0.064	0.001	-0.120	0.002	-0.002	0.001
Tenure (4)	0.26	0.626	0.551	0.683	-0.182	0.002	-0.176	0.002	-0.059	0.001	-0.117	0.002	-0.010	0.002
Marital status	0.35	0.709	0.648	0.756	-0.183	0.002	-0.170	0.002	-0.058	0.001	-0.113	0.002	-0.016	0.002
Extra salary	0.05	0.735	0.678	0.777	-0.186	0.002	-0.165	0.002	-0.055	0.002	-0.110	0.002	-0.021	0.002
Work permit	0.09	0.799	0.752	0.834	-0.186	0.002	-0.165	0.002	-0.055	0.002	-0.110	0.002	-0.021	0.002
Add-ons	0.07	0.820	0.777	0.851	-0.186	0.002	-0.163	0.002	-0.052	0.002	-0.111	0.003	-0.022	0.002
Hourly wage	0.04	0.828	0.789	0.857	-0.188	0.001	-0.164	0.002	-0.053	0.002	-0.111	0.002	-0.022	0.002
Over-time	0.03	0.842	0.806	0.869	-0.187	0.001	-0.167	0.002	-0.052	0.002	-0.115	0.002	-0.019	0.002
Share of full-time	0.01	0.893	0.877	0.905	-0.186	0.001	-0.173	0.003	-0.045	0.002	-0.128	0.003	-0.013	0.003
Wage bargaining (5)	0.01	0.901	0.886	0.913	-0.186	0.001	-0.173	0.003	-0.042	0.002	-0.131	0.003	-0.013	0.003

Notes: We use Supports 1–5 to conduct the sensitivity analysis of the unexplained gender pay gap estimates. Standard errors (SE) are calculated based on i.i.d. bootstrapping with 499 replications. All observations are weighted using their respective sample weights.

Table B.2: Common Support and Results from Exact Matching in the Public Sector

Variable added	Change in Adj. R^2 in %	Without support $S_i = 0$					With support $S_i = 1$						Difference to total raw gap	
			Share		Raw gap		Raw gap		Unexplained		Explained			
		Total	Women	Men	Δ	SE	Δ	SE	δ	SE	η	SE	ρ	SE
Management position	9.42	0	0	0	-	-	-0.139	0.002	-0.097	0.002	-0.042	0.001	0	0
Education	6.49	0	0	0	-	-	-0.139	0.003	-0.094	0.002	-0.044	0.002	0	0
Age (1)	8.21	0.000	0.000	0.000	0.214	0.175	-0.139	0.003	-0.079	0.002	-0.060	0.002	0.000	0.000
Industry	2.51	0.003	0.001	0.006	-0.528	0.096	-0.137	0.003	-0.060	0.002	-0.077	0.002	-0.001	0.000
Occupation	5.80	0.042	0.024	0.066	-0.267	0.013	-0.134	0.003	-0.055	0.002	-0.079	0.002	-0.005	0.001
Establishment size (2)	2.06	0.096	0.078	0.120	-0.234	0.011	-0.131	0.002	-0.044	0.002	-0.087	0.003	-0.008	0.001
Irregular payments	0.84	0.121	0.097	0.152	-0.224	0.010	-0.129	0.003	-0.042	0.002	-0.087	0.003	-0.009	0.001
Region	0.33	0.207	0.191	0.228	-0.223	0.009	-0.119	0.002	-0.035	0.001	-0.084	0.002	-0.020	0.002
Temporary contract (3)	1.10	0.218	0.204	0.238	-0.218	0.009	-0.119	0.002	-0.037	0.001	-0.082	0.002	-0.020	0.002
Tenure (4)	0.57	0.336	0.322	0.356	-0.214	0.006	-0.103	0.002	-0.037	0.001	-0.066	0.002	-0.036	0.002
Marital status	0.37	0.432	0.422	0.445	-0.205	0.005	-0.090	0.002	-0.037	0.001	-0.053	0.002	-0.049	0.003
Extra salary	0.35	0.440	0.431	0.452	-0.203	0.005	-0.090	0.002	-0.038	0.001	-0.052	0.002	-0.049	0.003
Work permit	0.21	0.494	0.490	0.500	-0.199	0.005	-0.081	0.002	-0.039	0.001	-0.042	0.002	-0.058	0.003
Add-ons	0.11	0.524	0.520	0.529	-0.198	0.005	-0.075	0.002	-0.038	0.001	-0.037	0.002	-0.064	0.003
Hourly wage	0.06	0.526	0.523	0.530	-0.197	0.004	-0.075	0.002	-0.038	0.001	-0.037	0.002	-0.064	0.003
Over-time	0.02	0.542	0.539	0.545	-0.195	0.004	-0.072	0.002	-0.038	0.001	-0.035	0.002	-0.066	0.002
Share of full-time	0.02	0.670	0.684	0.653	-0.181	0.004	-0.049	0.002	-0.036	0.001	-0.013	0.002	-0.089	0.003
Wage bargaining (5)	0.00	0.683	0.697	0.664	-0.180	0.003	-0.045	0.002	-0.034	0.001	-0.010	0.002	-0.094	0.003

Notes: We use Supports 1–5 to conduct the sensitivity analysis of the unexplained gender pay gap estimates. Standard errors (SE) are calculated based on i.i.d. bootstrapping with 499 replications. All observations are weighted using their respective sample weights.

Table B.3: Alternative Order of Variables

Sector-specific	Priv.	Sector-specific	Pub.	Random	Priv.	Pub.	Increasing importance	Priv.	Pub.
Management position	1.000	Management position	1.000	Temporary contract	1.000	1.000	Wage bargaining	1.000	1.000
Education	1.000	Age	1.000	Irregular payments	1.000	1.000	Share of full-time	1.000	1.000
Industry	1.000	Education	1.000	Occupation	1.000	1.000	Over-time	1.000	1.000
Age	0.999	Occupation	0.997	Age	1.000	1.000	Hourly wage	1.000	1.000
Occupation	0.951	Industry	0.976	Region	0.998	0.996	Add-ons	1.000	1.000
Irregular payments	0.924	Establishment size	0.922	Management position	0.991	0.987	Work permit	1.000	1.000
Establishment size	0.806	Temporary contract	0.911	Hourly wage	0.976	0.979	Extra salary	1.000	0.999
Region	0.625	Irregular payments	0.891	Establishment size	0.914	0.925	Marital status	1.000	0.997
Marital status	0.520	Tenure	0.795	Add-ons	0.896	0.911	Tenure	0.994	0.986
Tenure	0.361	Marital status	0.706	Extra salary	0.858	0.903	Temporary contract	0.991	0.982
Work permit	0.279	Extra salary	0.690	Work permit	0.780	0.867	Region	0.944	0.925
Add-ons	0.250	Region	0.569	Education	0.651	0.786	Irregular payments	0.916	0.904
Extra salary	0.229	Work permit	0.510	Over-time	0.616	0.766	Establishment size	0.795	0.799
Hourly wage	0.216	Add-ons	0.480	Marital status	0.502	0.680	Occupation	0.431	0.592
Over-time	0.199	Hourly wage	0.477	Wage bargaining	0.443	0.643	Industry	0.274	0.515
Wage bargaining	0.182	Over-time	0.461	Tenure	0.279	0.497	Age	0.182	0.401
Temporary contract	0.177	Share of full-time	0.316	Industry	0.177	0.438	Education	0.132	0.327
Share of full-time	0.114	Wage bargaining	0.303	Share of full-time	0.114	0.303	Management position	0.114	0.303

Notes: The sector-specific variants order the variables according to decreasing importance for explaining male wages in the respective sector. The random variant uses a random order. The increasing-importance variant reverses the order of variables in the main specification, i.e. order according to increasing importance for explaining man wages, where a weighted average of the private and public sector is used.

Table B.4: Trimmed Male Observations (Unweighted)

	Support				
	1	2	3	4	5
**Private Sector**					
Total male observations	641'035	508'326	375'608	275'861	88'346
Trimmed male observations for IPW and AIPW					
# observations	3'206	2'542	1'879	1'380	442
Percent	0.5%	0.5%	0.5%	0.5%	0.5%
**Public Sector**					
Total male observations	177'829	164'185	148'682	129'409	71'836
Trimmed male observations for IPW and AIPW					
# observations	890	821	744	648	360
Percent	0.5%	0.5%	0.5%	0.5%	0.5%

Notes: Supports 1-5 are defined in Table 4. We use trimming for IPW and AIPW. We trim male observations with importance weights above the 99.5% quantile.

Table B.5: Unweighted Number and Share of Women on Support for PSM and EXPSM

	Support				
	1	2	3	4	5
Private Sector					
Default	491'007	447'258	369'524	294'275	87'749
	100.0%	91.1%	75.3%	59.9%	17.9%
PSM baseline model	490'204	446'549	368'943	293'785	87'593
	99.8%	90.9%	75.1%	59.8%	17.8%
PSM full model	490'152	446'533	368'863	293'725	87'531
	99.8%	90.9%	75.1%	59.8%	17.8%
PSM ML model	490'133	446'515	368'890	293'770	87'583
	99.8%	90.9%	75.1%	59.8%	17.8%
EXPSM baseline model	490'204	444'013	366'570	291'751	87'000
	99.8%	90.4%	74.7%	59.4%	17.7%
EXPSM full model	490'152	443'982	366'522	291'773	87'027
	99.8%	90.4%	74.6%	59.4%	17.7%
EXPSM ML model	490'133	444'010	366'518	291'761	87'038
	99.8%	90.4%	74.6%	59.4%	17.7%
Public Sector					
Default	227'615	219'087	201'364	177'977	86'429
	100.0%	96.3%	88.5%	78.2%	38.0%
PSM baseline model	227'088	218'567	200'905	177'575	86'188
	99.8%	96.0%	88.3%	78.0%	37.9%
PSM full model	227'008	218'544	200'843	177'537	86'207
	99.7%	96.0%	88.2%	78.0%	37.9%
PSM ML model	227'073	218'583	200'866	177'557	86'201
	99.8%	96.0%	88.2%	78.0%	37.9%
EXPSM baseline model	227'088	217'783	200'078	176'658	85'823
	99.8%	95.7%	87.9%	77.6%	37.7%
EXPSM full model	227'088	217'755	199'894	176'628	85'836
	99.8%	95.7%	87.8%	77.6%	37.7%
EXPSM ML model	227'073	217'810	199'993	176'731	85'890
	99.8%	95.7%	87.9%	77.6%	37.7%

Notes: Supports 1-5 are defined in Table 4. The shares on support for the baseline samples differ from those reported in this table because sample weights are not applied here. We use the "default" sample of women for all estimators, except for PSM and EXPSM. For these two estimators, a few additional women are excluded, as no comparable men are observed within the matching radius. Therefore, the number of observations for PSM and EXPSM is reported separately in this table.

Table B.6: Average Characteristics of Women by Support Version in the Public Sector

Support	Mean 1	Mean 2	Std. Diff. 2 vs. 1	Mean 3	Std. Diff. 3 vs. 1	Mean 4	Std. Diff. 4 vs. 1	Mean 5	Std. Diff. 5 vs. 1
Standardised monthly wage (in CHF)	7915	7944	1.2	8011	4.0	8076	6.7	8427	21.2
Irregular payments (incl. bonuses)	0.148	0.146	0.4	0.134	4.1	0.122	7.5	0.092	17.2
Age	41.661	41.668	0.1	41.624	0.4	41.464	1.9	40.465	11.2
Education: University	0.363	0.367	0.8	0.378	3.1	0.395	6.5	0.489	25.6
Education: Vocational	0.424	0.420	0.7	0.410	2.7	0.395	5.8	0.312	23.3
Education: No vocational	0.066	0.064	0.6	0.058	3.2	0.054	5.0	0.028	18.0
Tenure	8.507	8.563	0.7	8.670	2.0	8.823	3.8	9.093	7.0
Management Level: Top	0.009	0.008	0.8	0.008	1.5	0.007	2.1	0.007	2.6
Management Level: Upper	0.081	0.081	0.1	0.083	0.9	0.087	2.2	0.130	16.1
Management Level: Middle	0.058	0.055	1.3	0.053	2.4	0.048	4.5	0.035	11.2
Management Level: Lower	0.051	0.047	2.1	0.043	3.9	0.038	6.5	0.022	15.5
Management Level: None	0.724	0.730	1.3	0.730	1.4	0.731	1.5	0.679	9.9
Occupation: Managers	0.045	0.043	1.4	0.040	2.7	0.036	4.9	0.025	11.3
Occupation: Professionals	0.429	0.437	1.5	0.454	5.0	0.480	10.2	0.646	44.5
Occupation: Technicians and Associate Professionals	0.296	0.297	0.1	0.292	1.0	0.285	2.4	0.216	18.5
Occupation: Clerical Support Workers	0.069	0.067	1.0	0.062	2.9	0.053	7.0	0.025	21.1
Occupation: Services and Sales Workers	0.107	0.109	0.7	0.111	1.4	0.109	0.7	0.064	15.3
Occupation: Craft and Related Trades Workers	0.015	0.016	0.3	0.016	0.8	0.016	0.6	0.011	3.8
Occupation: Plant/Machine Operators, Assemblers	0.004	0.003	0.8	0.003	0.8	0.003	0.5	0.003	0.4
Occupation: Elementary Occupations	0.034	0.029	2.8	0.022	7.4	0.018	10.1	0.010	16.1
Part-time	0.699	0.697	0.3	0.694	1.0	0.691	1.6	0.604	20.0
Industry: Less knowledge-intensive services	0.005	0.005	0.6	0.004	0.8	0.004	0.9	0.004	1.6
Industry: Knowledge-intensive services	0.987	0.989	2.2	0.990	3.1	0.991	4.6	0.995	8.3
Industry: Other (incl. construction)	0.008	0.006	2.4	0.005	3.4	0.004	5.3	0.001	9.9
Firm size: <20	0.004	0.001	4.3	0.001	6.6	0.000	7.7	0.000	8.4
Firm size: 20–49	0.006	0.003	4.5	0.001	7.4	0.001	9.1	0.000	10.5
Firm size: 50–249	0.051	0.045	3.1	0.036	7.2	0.027	12.5	0.005	28.1
Firm size: 250–999	0.070	0.065	2.1	0.056	5.9	0.045	10.8	0.021	23.3
Firm size: ≥1000	0.870	0.886	5.2	0.906	11.6	0.927	19.3	0.973	39.3
# Observed Women	227,615	219,087		201,364		177,977		86,429	

Notes: The table shows mean characteristics of women across support versions 1 to 5 (see Table 4). Standardised differences (Std. Diff.) are relative to support version 1. Monthly wages are standardised to 100% full-time equivalents and exclude overtime hours.

C Machine learning model (ML)

Instead of manually choosing which variables to include in a model, machine learning (ML) methods can automatically find a good balance between fitting the data well and avoiding overcomplicating the model. This is especially helpful when working with smaller datasets, where adding too many details can easily lead to misleading results. In our analysis, we use a popular ML technique called the Lasso to build these models. Exactly how we apply the Lasso depends on the specific estimation method we are using.

The Lasso is a penalised regression method designed to achieve a good balance between model complexity and predictive accuracy (for a detailed description, see Hastie et al. (2009)). It does so by shrinking the estimated coefficients of some control variables towards zero. When a coefficient becomes exactly zero, the corresponding variable is effectively excluded from the model. Hence, the Lasso simultaneously estimates the model and selects the relevant variables.

Specifically, to predict men's wages $\hat{\mu}_0(x)$ in the baseline and full models for BO, the Lasso solves the following optimization problem:

$$(\hat{\alpha}_0, \hat{\beta}_0) = \arg\min_{a,b} \frac{1}{N} \sum_{i=1}^{N} (Y_i - a - X_i b)^2 + \lambda |b|_1, \tag{C.1}$$

where $|b|_1 = \sum_{j=1}^{p} |b_j|$ is the ℓ_1-norm of the coefficient vector b, and $\lambda \geq 0$ is the tuning parameter that determines the strength of the penalty.

If $\lambda = 0$, the Lasso reduces to standard OLS and includes all covariates. As λ increases, more coefficients are shrunk towards zero, and some variables are dropped from the model. In the extreme case where $\lambda \to \infty$, all coefficients except for the intercept $\hat{\alpha}_0$ are exactly zero.

We determine the optimal value of λ through a five-fold cross-validation procedure (Chetverikov et al., 2021). That is, we split the sample into five subsets, estimate the model on four subsets, and validate it on the remaining subset, rotating this procedure across all folds. We then select the value of λ that minimizes the mean squared error (MSE) across the validation sets. To further improve model robustness, we apply the one-standard-error rule, which chooses the most parsimonious model whose MSE is within one standard error of the minimum (see, e.g., Hastie et al., 2009).

C.1 Differences across estimators

Having described the general principles of the ML model specification using Lasso, we now outline how its implementation differs across estimators. The estimators used in our

analysis require either a wage model or a propensity score model (i.e., the probability that an individual belongs to the group of women) as input. In traditional analysis, simple methods such as OLS and Logit models are employed to estimate these models. In our ML-based approach, we replace these traditional methods with more flexible techniques, namely Lasso or Logit-Lasso. However, the exact implementation varies slightly depending on the estimator, as different procedures are recommended in the literature.

To implement ML for the LRM, we apply the post-double-selection (PDS) procedure proposed by Belloni et al. (2013, 2014) (see Section C.1.1). For the BO, we use the T-learner approach as described by Künzel et al. (2019) (see Section C.1.2). For the AIPW estimator, we combine ML estimation with cross-fitting to ensure robustness and $\sqrt{N}$-consistency (see Section C.1.3).

For the PSM and EXPSM estimators, we estimate the propensity scores using Logit-Lasso (see Hastie et al., 2016, for an introduction to Logit-Lasso). In fact, we always use Logit-Lasso for propensity score estimation in the ML models, except in the PDS procedure, where a linear Lasso model is used to estimate the propensity score, following Belloni et al. (2013).

C.1.1 Post-double-selection (PDS) procedure for the LRM

To implement ML for the LRM, we adopt the post-double-selection (PDS) procedure proposed by Belloni et al. (2013, 2014). Applying the conventional Lasso directly to the LRM

$$Y_i = \alpha + G_i \delta_{LRM} + X_i \beta + \varepsilon_i, \tag{C.2}$$

risks omitting wage determinants that are highly correlated with the gender dummy G_i. As a result, important factors explaining gender differences could be inadvertently excluded, potentially leading to inaccurate estimates of the gender differences.

The PDS procedure circumvents this problem by selecting the union of variables that are either important predictors of wages or strongly correlated with gender. It involves three main steps:

First, the Lasso selects the relevant control variables in the linear wage equation

$$Y_i = \alpha_Y + X_i \beta_Y + \nu_i,$$

which does not include the gender dummy G_i. In contrast to equation (C.1), the entire sample of men and women is used at this stage. In practice, the Lasso may shrink some coefficients in β_Y to exactly zero, effectively excluding the corresponding control variables from the model.

Second, the Lasso selects the relevant dimensions of X_i in the linear gender model

$$G_i = \alpha_G + X_i\beta_G + \eta_i,$$

identifying those variables most strongly correlated with gender. Again, some coefficients in β_G may be shrunk to zero.

Third, we define $\tilde{X}i$ as the union of all variables selected in either the wage or gender model. We then estimate the final OLS model (termed 'post-Lasso'):

$$Y_i = \alpha_{PDS} + G_i\delta_{PDS} + \tilde{X}_i\beta_{PDS} + \tilde{\varepsilon}_i.$$

Belloni et al. (2013) show that the estimator δ_{PDS} is consistent and asymptotically normal. The PDS procedure permits the inclusion of high-dimensional control variables (i.e., more variables than observations), provided that the selected set $\tilde{X}_i$ remains sufficiently sparse. Furthermore, the procedure offers a form of double robustness, ensuring validity even if either the wage equation or the propensity score is misspecified.

C.1.2 T-learner for BO

As with the LRM, applying the Lasso naively within the Blinder–Oaxaca (BO) decomposition risks inaccurate estimates of $\hat{\delta}_{BO}$. However, the BO decomposition offers a convenient structure that allows the integration of machine learning techniques in a way that preserves consistency.

Specifically, we use the Lasso to estimate the conditional expected wage for men, $\hat{\mu}_0(x)$. This is unproblematic, as it serves purely as a prediction model for Y_i. The gender dummy G_i is not included in the wage equation for men, because it is redundant. The resulting $\hat{\mu}_0(x)$ is then used as a plug-in estimator within the BO decomposition:

$$\hat{\delta}_{BO} = \frac{1}{N_1}\sum_{i=1}^{N} G_i\left(Y_i - \hat{\mu}_0(X_i)\right). \tag{C.3}$$

In this framework, only the prediction of male wages needs to be modelled flexibly, the decomposition itself remains unchanged. This approach is referred to as the T-learner (Künzel et al., 2019).

C.1.3 Cross-fitting for AIPW

When applying the AIPW with ML, it is important to separate the data used for ML model training from the data used for estimating gender differences. Otherwise, the estimated gender differences may be inaccurate and imprecise. To address this concern and to achieve

$\sqrt{N}$-consistency, it is necessary to estimate the ML models for $\hat{\mu}_0(x)$ and $\hat{p}(x)$ on a different sample than the one used to compute $\hat{\delta}_{AIPW}$ (see Chernozhukov et al., 2018). We implement this approach through cross-fitting. Specifically, we partition the sample into two equally sized subsets. We first estimate $\hat{\mu}_0(x)$ and $\hat{p}(x)$ using the first subset, extrapolate the fitted values to the second subset, and then use these values to estimate $\hat{\delta}_{AIPW}$. We then switch the roles of the two subsets, repeating the procedure so that all observations are used efficiently. Finally, we report the average of the two estimates of $\hat{\delta}_{AIPW}$ across the partitions.

C.2 Selected variables

The ML models select among all control variables of the full model. Table C.1 documents the number of selected control variables for all ML models we consider. The final specifications vary by support and estimation procedure. For the wage equation, the ML model considers between 371 and 513 control variables for the private sector and between 215 and 306 control variables for the public sector. For the propensity score model, the ML model considers between 141 and 428 control variables for the private sector and between 126 and 230 control variables for the public sector.

C.3 Performance.

In Tables C.2 and C.3, we report the out-of-sample prediction power of the different nuisance parameter models using the full sample.

To obtain the out-of-sample prediction power, we use a cross-fitting procedure. This means, we partition the data into two equally sized samples, using one partition as a training sample and the other as a test sample. Thereafter, we switch the two partitions and report the average prediction power across the two samples.

The prediction power of the baseline, full and ML models do not differ strongly. Accordingly, the baseline model already explains a significant amount of the variation in the data. The prediction power of the full model is systematically better than the prediction power of the baseline model, but the additional gain is moderate. The ML model cannot systematically outperform the full model. The prediction power of the full and ML models is fairly similar, even though the ML model controls for many fewer variables than the full model. This suggests that the full model is too flexible. However, because of our large data set, the prediction power of the full model does not deteriorate compared to the ML model. The degrees of freedom loss of the full model compared to the ML model appear to be of minor importance. However, we expect that the ML model would outperform the full model in smaller samples.

Table C.1: Number of Included Covariates

	Support				
	1	2	3	4	5
**Private Sector**					
Baseline model	117	117	117	117	117
Full model	615	615	615	615	615
ML models					
Wage equation	425	456	464	513	384
Propensity-score model	423	428	386	314	222
AIPW ML models					
Wage equation (fold 1)	475	456	371	409	394
Wage equation (fold 2)	469	438	454	458	383
Propensity-score model (fold 1)	319	320	276	275	169
Propensity-score model (fold 2)	377	335	394	265	172
PDS ML model					
Wage equation	462	463	470	473	477
Propensity-score model	401	346	344	277	141
Union (Post-LASSO)	539	532	542	518	496
**Public Sector**					
Baseline model	74	74	74	74	74
Full model	396	396	396	396	396
ML models					
Wage equation	241	241	283	306	292
Propensity-score model	189	213	209	170	196
AIPW ML models					
Wage equation (fold 1)	234	297	257	280	243
Wage equation (fold 2)	215	206	267	285	285
Propensity-score model (fold 1)	185	141	137	145	126
Propensity-score model (fold 2)	197	151	143	186	202
PDS ML model					
Wage equation	238	248	283	305	289
Propensity-score model	230	184	191	228	186
Union (Post-LASSO)	319	303	326	341	327

Notes: We use the full sample and supports 1–5 as defined in Table 4. In the baseline and full model we fix the number of included covariates. In The ML models we use LASSO estimators to select the included covariates in a data-driven way. Accordingly, the number of included covariates of ML models differ by estimator and support. For BO, we use all male observations to estimate the wage equation with LASSO. For IPW, PSM, and EXPSM, we use all observations to estimate the propensity score model with Logit-LASSO. For AIPW, we use a two-fold cross-fitting procedure to estimate the ML models (see, e.g., Chernozhukov et al., 2018). Therefore, we estimate separate wage equations and propensity score models in each fold. For post-double-selection (PDS) procedure, we use all observations to estimate the wage equation (man and woman observations) and we implement the propensity score model with linear LASSO instead of Logit-LASSO. Finally, we control for the union of all covariates selected in either the wage equation or the propensity score model in the structural Post-LASSO model (see, e.g., Belloni et al., 2013, for a description of the PDS procedure).

Table C.2: Out-of-Sample Model Fit in the Private Sector

	Support				
	1	2	3	4	5
**Wage Equation**					
Baseline model					
MSE	0.062	0.065	0.065	0.063	0.059
R-squared	0.646	0.649	0.665	0.675	0.732
Full model					
MSE	0.055	0.057	0.056	0.054	0.049
R-squared	0.690	0.690	0.710	0.720	0.776
ML model					
MSE	0.055	0.058	0.057	0.055	0.049
R-squared	0.689	0.687	0.706	0.718	0.774
**Propensity Score Model**					
Baseline model					
MSE	0.149	0.164	0.180	0.190	0.228
R-squared	0.393	0.340	0.279	0.239	0.087
Classification Error	0.214	0.238	0.266	0.284	0.379
Full model					
MSE	0.145	0.161	0.177	0.187	0.227
R-squared	0.408	0.354	0.291	0.250	0.092
Classification Error	0.209	0.234	0.263	0.282	0.380
ML model					
MSE	0.145	0.161	0.177	0.188	0.226
R-squared	0.407	0.353	0.290	0.249	0.097
Classification Error	0.210	0.235	0.264	0.281	0.377

Notes: We use the full sample and supports 1–5 as defined in Table 4. For all models, we use a two-fold cross-fitting procedure to estimate the out-of-sample model fit. The reported ML models correspond to the AIPW ML models.

Table C.3: Out-of-Sample Model Fit in the Public Sector

	Support				
	1	2	3	4	5
**Wage Equation**					
Baseline model					
MSE	0.040	0.041	0.040	0.040	0.036
R-squared	0.643	0.644	0.645	0.653	0.694
Full model					
MSE	0.035	0.035	0.034	0.033	0.029
R-squared	0.688	0.691	0.698	0.708	0.753
ML model					
MSE	0.036	0.036	0.035	0.034	0.029
R-squared	0.686	0.687	0.695	0.706	0.750
**Propensity Score Model**					
Baseline model					
MSE	0.169	0.174	0.180	0.186	0.214
R-squared	0.311	0.285	0.261	0.236	0.136
Classification Error	0.242	0.251	0.262	0.271	0.330
Full model					
MSE	0.166	0.171	0.177	0.182	0.210
R-squared	0.324	0.296	0.273	0.251	0.152
Classification Error	0.238	0.247	0.257	0.268	0.328
ML model					
MSE	0.166	0.172	0.178	0.183	0.211
R-squared	0.321	0.294	0.271	0.248	0.151
Classification Error	0.239	0.248	0.259	0.268	0.329

Notes: We use the full sample and supports 1–5 as defined in Table 4. For all models, we use a two-fold cross-fitting procedure to estimate the out-of-sample model fit. The reported ML models correspond to the AIPW ML models.

D Full Estimation Results

Table D.1: Average Unexplained Gender Pay Gap in the Private Sector

	Support				
	1	2	3	4	5
Raw Difference	-0.186	-0.180	-0.184	-0.176	-0.173
	(0.001)	(0.001)	(0.001)	(0.001)	(0.0005)
LRM baseline model	-0.100	-0.093	-0.087	-0.081	-0.068
	(0.001)	(0.001)	(0.001)	(0.001)	(0.0003)
LRM full model	-0.089	-0.084	-0.078	-0.073	-0.058
	(0.001)	(0.001)	(0.001)	(0.001)	(0.0002)
LRM ML model (PDS)	-0.089	-0.084	-0.078	-0.073	-0.058
	(0.001)	(0.001)	(0.001)	(0.001)	(0.0002)
BO baseline model	-0.077	-0.073	-0.073	-0.072	-0.062
	(0.001)	(0.002)	(0.002)	(0.001)	(0.002)
BO full model	-0.076	-0.071	-0.069	-0.066	-0.052
	(0.001)	(0.002)	(0.002)	(0.001)	(0.002)
BO ML model	-0.076	-0.072	-0.070	-0.066	-0.055
	(0.001)	(0.002)	(0.002)	(0.001)	(0.002)
IPW baseline model	-0.068	-0.064	-0.059	-0.053	-0.066
	(0.002)	(0.002)	(0.002)	(0.002)	(0.002)
IPW full model	-0.069	-0.062	-0.057	-0.050	-0.060
	(0.002)	(0.002)	(0.002)	(0.003)	(0.004)
IPW ML model	-0.070	-0.064	-0.061	-0.056	-0.069
	(0.002)	(0.002)	(0.002)	(0.002)	(0.003)
AIPW baseline model	-0.074	-0.074	-0.076	-0.072	-0.063
	(0.002)	(0.002)	(0.002)	(0.002)	(0.002)
AIPW full model	-0.072	-0.068	-0.068	-0.065	-0.052
	(0.001)	(0.002)	(0.002)	(0.001)	(0.002)
AIPW ML model	-0.072	-0.069	-0.068	-0.065	-0.055
	(0.001)	(0.001)	(0.002)	(0.001)	(0.002)
EXM	-0.131	-0.069	-0.064	-0.059	-0.042
	(0.001)	(0.001)	(0.001)	(0.001)	(0.002)
PSM baseline model	-0.069	-0.064	-0.060	-0.056	-0.058
	(0.002)	(0.002)	(0.002)	(0.002)	(0.002)
PSM full model	-0.068	-0.064	-0.059	-0.054	-0.053
	(0.002)	(0.002)	(0.002)	(0.005)	(0.002)
PSM ML model	-0.068	-0.064	-0.059	-0.055	-0.054
	(0.002)	(0.002)	(0.002)	(0.002)	(0.002)
EXPSM baseline model	-0.069	-0.065	-0.059	-0.056	-0.042
	(0.002)	(0.002)	(0.001)	(0.001)	(0.002)
EXPSM full model	-0.068	-0.065	-0.060	-0.056	-0.041
	(0.002)	(0.002)	(0.001)	(0.002)	(0.002)
EXPSM ML model	-0.068	-0.065	-0.059	-0.056	-0.042
	(0.002)	(0.002)	(0.001)	(0.001)	(0.002)

Notes: Supports 1-5 are defined in Table 4. The gender pay gap is in all specifications statistically significant on the 1%-level. Standard errors are in parentheses. BO, IPW, EXM, PSM, and EXPSM standard errors are bootstrapped using a non-parametric i.i.d. bootstrap with 499 resamples. For LRM and AIPW we estimate the asymptotic standard errors. PDS is the abbreviation for post-double-selection procedure.

Table D.2: Average Unexplained Gender Pay Gap in the Public Sector

	Support				
	1	2	3	4	5
Raw Difference	-0.139	-0.131	-0.119	-0.103	-0.045
	(0.003)	(0.002)	(0.002)	(0.002)	(0.001)
LRM baseline model	-0.067	-0.064	-0.063	-0.059	-0.047
	(0.002	(0.001)	(0.001)	(0.001)	(0.0004)
LRM full model	-0.059	-0.057	-0.056	-0.052	-0.042
	(0.002	(0.001)	(0.001)	(0.001)	(0.0004)
LRM ML model (PDS)	-0.059	-0.058	-0.056	-0.052	-0.042
	(0.002	(0.001)	(0.001)	(0.001)	(0.0004)
BO baseline model	-0.064	-0.063	-0.061	-0.057	-0.045
	(0.003)	(0.002)	(0.002)	(0.002)	(0.001)
BO full model	-0.051	-0.051	-0.048	-0.046	-0.039
	(0.002)	(0.002)	(0.002)	(0.001)	(0.001)
BO ML model	-0.052	-0.052	-0.049	-0.046	-0.040
	(0.002)	(0.002)	(0.002)	(0.001)	(0.001)
IPW baseline model	-0.065	-0.059	-0.056	-0.051	-0.033
	(0.005)	(0.003)	(0.003)	(0.002)	(0.001)
IPW full model	-0.063	-0.058	-0.054	-0.048	-0.030
	(0.003)	(0.003)	(0.002)	(0.002)	(0.001)
IPW ML model	-0.067	-0.061	-0.055	-0.051	-0.033
	(0.003)	(0.003)	(0.003)	(0.003)	(0.002)
AIPW baseline model	-0.064	-0.060	-0.058	-0.055	-0.045
	(0.003)	(0.002)	(0.002)	(0.002)	(0.001)
AIPW full model	-0.051	-0.051	-0.049	-0.047	-0.040
	(0.002)	(0.002)	(0.002)	(0.001)	(0.001)
AIPW ML model	-0.054	-0.052	-0.049	-0.046	-0.041
	(0.003)	(0.002)	(0.002)	(0.001)	(0.001)
EXM	-0.079	-0.044	-0.037	-0.037	-0.034
	(0.002)	(0.002)	(0.001)	(0.001)	(0.001)
PSM baseline model	-0.052	-0.047	-0.043	-0.038	-0.036
	(0.002)	(0.002)	(0.002)	(0.002)	(0.001)
PSM full model	-0.047	-0.044	-0.038	-0.036	-0.034
	(0.002)	(0.002)	(0.002)	(0.002)	(0.001)
PSM ML model	-0.048	-0.044	-0.041	-0.037	-0.037
	(0.002)	(0.002)	(0.002)	(0.002)	(0.001)
EXPSM baseline model	-0.052	-0.036	-0.032	-0.034	-0.033
	(0.002)	(0.002)	(0.001)	(0.001)	(0.001)
EXPSM full model	-0.047	-0.037	-0.032	-0.033	-0.033
	(0.002)	(0.002)	(0.001)	(0.001)	(0.001)
EXPSM ML model	-0.048	-0.037	-0.032	-0.033	-0.032
	(0.002)	(0.002)	(0.001)	(0.001)	(0.001)

Notes: Supports 1-5 are defined in Table 4. The gender pay gap is in all specifications statistically significant on the 1%-level. Standard errors are in parentheses. BO, IPW, EXM, PSM, and EXPSM standard errors are bootstrapped using a non-parametric i.i.d. bootstrap with 499 resamples. For LRM and AIPW we estimate the asymptotic standard errors. PDS is the abbreviation for post-double-selection procedure.

Table D.3: Average Unexplained Gender Pay Gap in the Private Sector (10,000 Observations)

	Support				
	1	2	3	4	5
Raw Difference	-0.1843	-0.1292	-0.1396	-0.1048	-0.0532
	(0.0140)	(0.0220)	(0.0252)	(0.0306)	(0.0535)
LRM baseline model	-0.0984	-0.0708	-0.0570	-0.0464	-0.0253
	(0.0115)	(0.0142)	(0.0167)	(0.0199)	(0.0411)
LRM full model	-0.0867	-0.0655	-0.0506	-0.0430	-0.0249
	(0.0101)	(0.0127)	(0.0157)	(0.0197)	(0.0443)
LRM ML model (PDS)	-0.0878	-0.0669	-0.0538	-0.0441	-0.0266
	(0.0104)	(0.0132)	(0.0149)	(0.0189)	(0.0391)
BO baseline model	-0.0752	-0.0678	-0.0543	-0.0438	-0.0353
	(0.0151)	(0.0188)	(0.0212)	(0.0260)	(1.4129)
BO full model	-0.0744	-0.0638	-0.0446	-0.0341	275.4652
	(0.0155)	(0.0285)	(0.1141)	(0.1684)	(4285.2360)
BO ML model	-0.0966	-0.0793	-0.0728	-0.0622	-0.0436
	(0.0133)	(0.0153)	(0.0176)	(0.0223)	(0.0458)
IPW baseline model	-0.0719	-0.0532	-0.0421	-0.0300	-0.0293
	(0.0192)	(0.0216)	(0.0257)	(0.0346)	(0.0747)
IPW full model	-0.0818	-0.0714	-0.0932	-0.0482	-0.0321
	(0.0172)	(0.0412)	(0.0487)	(0.0674)	(0.1172)
IPW ML model	-0.1066	-0.0939	-0.1016	-0.0921	-0.0532
	(0.0157)	(0.0205)	(0.0260)	(0.0317)	(0.0535)
AIPW baseline model	-0.0761	-0.0697	-0.0560	-0.0451	-0.0355
	(0.0176)	(0.0198)	(0.0237)	(0.0292)	(1.4129)
AIPW full model	-0.0713	-0.0645	-0.0449	-0.0345	275.7378
	(0.0178)	(0.0298)	(0.1142)	(0.1685)	(4287.3750)
AIPW ML model	-0.0802	-0.0701	-0.0660	-0.0616	-0.0434
	(0.0179)	(0.0207)	(0.0238)	(0.0320)	(0.0863)
EXM	-0.1347	-0.0625	-0.0499	-0.0447	-0.0275
	(0.0110)	(0.0146)	(0.0174)	(0.0209)	(0.0411)
PSM baseline model	-0.0908	-0.0678	-0.0597	-0.0505	-0.0280
	(0.0141)	(0.0181)	(0.0204)	(0.0258)	(0.0455)
PSM full model	-0.0980	-0.0811	-0.0795	-0.0582	-0.0299
	(0.0141)	(0.0211)	(0.0308)	(0.0308)	(0.0603)
PSM ML model	-0.0861	-0.0628	-0.0547	-0.0523	-0.0319
	(0.0142)	(0.0189)	(0.0220)	(0.0267)	(0.0434)
EXPSM baseline model	-0.0908	-0.0616	-0.0480	-0.0441	-0.0266
	(0.0141)	(0.0148)	(0.0176)	(0.0209)	(0.0419)
EXPSM full model	-0.0980	-0.0594	-0.0392	-0.0392	-0.0248
	(0.0141)	(0.0171)	(0.0242)	(0.0258)	(0.0517)
EXPSM ML model	-0.0861	-0.0616	-0.0485	-0.0444	-0.0274
	(0.0142)	(0.0148)	(0.0174)	(0.0210)	(0.0411)

Notes: The estimates report the average unexplained gender pay gap in the private sector. They are based on averages from 1,000 random draws of 10,000 observations from the full sample. Standard errors (in parentheses) are simulated using the same resampling procedure. Supports 1–5 define different sets of covariates used for estimation (see Table 4). PDS refers to the post-double-selection procedure.

Table D.4: Average Unexplained Gender Pay Gap in the Private Sector (100,000 Observations)

	Support				
	1	2	3	4	5
Raw Difference	-0.1858	-0.1596	-0.1659	-0.1499	-0.1165
	(0.0041)	(0.0055)	(0.0061)	(0.0068)	(0.0124)
LRM baseline model	-0.0994	-0.0856	-0.0755	-0.0667	-0.0411
	(0.0036)	(0.0041)	(0.0043)	(0.0047)	(0.0073)
LRM full model	-0.0889	-0.0780	-0.0675	-0.0597	-0.0362
	(0.0036)	(0.0038)	(0.0038)	(0.0041)	(0.0066)
LRM ML model (PDS)	-0.0890	-0.0782	-0.0677	-0.0599	-0.0381
	(0.0036)	(0.0039)	(0.0039)	(0.0042)	(0.0067)
BO baseline model	-0.0769	-0.0746	-0.0678	-0.0625	-0.0378
	(0.0049)	(0.0052)	(0.0054)	(0.0058)	(0.0075)
BO full model	-0.0754	-0.0701	-0.0614	-0.0563	-0.0338
	(0.0050)	(0.0052)	(0.0051)	(0.0055)	(0.0071)
BO ML model	-0.0779	-0.0749	-0.0670	-0.0632	-0.0472
	(0.0044)	(0.0049)	(0.0053)	(0.0057)	(0.0086)
IPW baseline model	-0.0675	-0.0567	-0.0468	-0.0459	-0.0445
	(0.0060)	(0.0063)	(0.0062)	(0.0064)	(0.0082)
IPW full model	-0.0676	-0.0565	-0.0471	-0.0432	-0.0441
	(0.0055)	(0.0058)	(0.0059)	(0.0061)	(0.0083)
IPW ML model	-0.0781	-0.0702	-0.0650	-0.0661	-0.0976
	(0.0055)	(0.0058)	(0.0061)	(0.0075)	(0.0140)
AIPW baseline model	-0.0806	-0.0789	-0.0721	-0.0648	-0.0386
	(0.0066)	(0.0056)	(0.0058)	(0.0059)	(0.0076)
AIPW full model	-0.0745	-0.0702	-0.0619	-0.0563	-0.0338
	(0.0071)	(0.0060)	(0.0055)	(0.0057)	(0.0071)
AIPW ML model	-0.0746	-0.0712	-0.0648	-0.0601	-0.0481
	(0.0060)	(0.0057)	(0.0058)	(0.0061)	(0.0094)
EXM	-0.1312	-0.0690	-0.0579	-0.0524	-0.0342
	(0.0033)	(0.0040)	(0.0041)	(0.0045)	(0.0070)
PSM baseline model	-0.0747	-0.0652	-0.0559	-0.0513	-0.0415
	(0.0051)	(0.0051)	(0.0056)	(0.0060)	(0.0088)
PSM full model	-0.0737	-0.0653	-0.0553	-0.0496	-0.0387
	(0.0054)	(0.0052)	(0.0053)	(0.0058)	(0.0086)
PSM ML model	-0.0725	-0.0617	-0.0508	-0.0424	-0.0431
	(0.0053)	(0.0052)	(0.0056)	(0.0065)	(0.0103)
EXPSM baseline model	-0.0747	-0.0662	-0.0550	-0.0505	-0.0338
	(0.0051)	(0.0041)	(0.0042)	(0.0047)	(0.0071)
EXPSM full model	-0.0737	-0.0668	-0.0556	-0.0507	-0.0337
	(0.0054)	(0.0042)	(0.0042)	(0.0047)	(0.0071)
EXPSM ML model	-0.0725	-0.0659	-0.0552	-0.0508	-0.0340
	(0.0053)	(0.0042)	(0.0042)	(0.0047)	(0.0070)

Notes: The estimates report the average unexplained gender pay gap in the private sector. They are based on averages from 500 random draws of 100,000 observations from the full sample. Standard errors (in parentheses) are simulated using the same resampling procedure. Supports 1–5 define different sets of covariates used for estimation (see Table 4). PDS refers to the post-double-selection procedure.

Table D.5: Average Characteristics of Women on Support in the Private Sector (100,000 Observations)

	Support 1		Support 2		Support 3		Support 4		Support 5	
	Mean	% Diff.	Mean	% Diff.	Mean	% Diff.	Mean	% Diff.	Mean	% Diff.
Standardised monthly wage (in CHF)	6533	-0.0	6408	-1.5	6425	-1.2	6335	-1.8	6362	-1.5
Irregular payments (incl. bonuses)	.397	-0.1	.396	-0.5	.391	-1.8	.392	-0.3	.442	1.8
Age	40.2	0.0	40.0	-0.2	40.0	0.0	39.3	-0.8	36.6	-1.3
Education: University	.145	0.1	.127	-9.4	.119	-11.9	.115	-13.0	.125	-8.5
Education: Vocational	.574	-0.1	.56	-2.0	.525	-5.4	.49	-8.5	.444	-6.9
Education: No vocational	.191	0.0	.203	4.1	.208	3.9	.216	5.3	.195	-0.2
Management Level: Top	.015	2.6	.008	-36.7	.004	-57.0	.002	-59.5	.002	-37.6
Management Level: Upper	.041	0.3	.03	-19.2	.028	-14.1	.028	-8.2	.038	10.9
Management Level: Middle	.068	0.3	.05	-17.8	.048	-14.8	.045	-13.6	.042	-17.9
Management Level: Lower	.066	0.4	.044	-23.3	.043	-19.6	.039	-19.2	.049	-3.4
Management Level: None	.809	0.0	.868	4.3	.877	3.2	.886	2.5	.869	1.0
Part-time	.552	0.0	.559	1.4	.566	3.1	.565	3.0	.295	-8.2
Tenure	7.2	0.0	7.4	2.3	8.0	6.2	8.3	10.3	8.8	9.7
Industry: Low-tech manufacturing	.065	-0.5	.051	-15.8	.039	-24.5	.031	-31.0	.026	-30.5
Industry: High-tech manufacturing	.09	0.4	.081	-8.5	.077	-12.1	.073	-10.6	.083	-5.4
Industry: Less knowledge-intensive services	.377	-0.1	.407	6.0	.458	13.8	.524	23.3	.618	17.1
Industry: Knowledge-intensive services	.454	0.0	.452	-0.6	.421	-6.3	.367	-16.6	.268	-21.3
Industry: Other (incl. construction)	.014	2.0	.009	-27.6	.006	-32.7	.005	-34.9	.004	-27.6
Firm size: $¡$ 20	.074	0.1	.031	-45.1	.013	-58.9	.009	-55.5	.005	-45.8
Firm size: 20–49	.065	0.7	.034	-37.0	.013	-62.5	.008	-65.0	.005	-58.4
Firm size: 50–249	.247	0.0	.24	-3.1	.185	-20.4	.138	-34.1	.063	-44.4
Firm size: 250–999	.203	-0.1	.202	-1.6	.171	-16.4	.136	-30.0	.105	-31.5
Firm size: $\geq$ 1000	.411	-0.1	.493	13.0	.617	24.7	.709	28.4	.822	15.4
Occupation: Managers	.076	0.4	.053	-22.7	.054	-14.1	.055	-7.4	.081	8.1
Occupation: Professionals	.132	-0.1	.118	-9.5	.103	-18.0	.095	-18.7	.106	-5.6
Occupation: Technicians and Associate Professionals	.261	0.1	.265	-0.7	.241	-8.8	.197	-22.5	.134	-30.7
Occupation: Clerical Support Workers	.109	0.4	.07	-25.2	.061	-17.9	.069	6.2	.129	42.3
Occupation: Services and Sales Workers	.269	0.0	.339	18.9	.385	21.5	.415	18.8	.337	2.2
Occupation: Craft and Related Trades Workers	.029	0.2	.027	-8.4	.025	-12.1	.021	-21.9	.018	-27.1
Occupation: Plant/Machine Operators, Assemblers	.022	1.5	.021	-8.2	.018	-21.8	.015	-28.1	.016	-25.3
Occupation: Elementary Occupations	.101	-0.4	.108	4.7	.114	8.8	.133	23.4	.179	16.8
# Observed Women	43360		30560		19646		12128		2091	

Notes: The table reports mean characteristics of women across support versions 1 to 5 (see Table 4). Mean values are calculated as averages over 500 randomly drawn subsamples of 100,000 observations from the full sample. In expectation, subsample means would match the full sample means if the support did not deteriorate more rapidly in smaller subsamples. Percentage differences (% Diff.) are relative to the mean characteristics in the full sample with the same support version.

Table D.6: Average Characteristics of Women on Support in the Private Sector (10,000 Observations)

	Support 1		Support 2		Support 3		Support 4		Support 5	
	Mean	% Diff.	Mean	% Diff.	Mean	% Diff.	Mean	% Diff.	Mean	% Diff.
Standardised monthly wage (in CHF)	6531	0.0	6181	-5.0	62447	-3.9	6132	-5.0	6268	-2.9
Irregular payments (incl. bonuses)	.397	0.0	.38	-4.6	.389	-2.2	.405	2.9	.457	5.4
Age	40.2	0.0	40.0	-0.3	40.0	0.0	39.3	-0.9	36.6	-1.3
Education: University	.145	0.1	.096	-31.5	.097	-28.4	.091	-31.4	.116	-15.6
Education: Vocational	.574	-0.1	.553	-3.2	.457	-17.7	.412	-23.0	.43	-9.8
Education: No vocational	.191	0.1	.203	3.9	.233	16.4	.232	13.4	.163	-16.2
Management Level: Top	.015	0.1	.003	-73.3	.002	-80.0	.001	-79.1	.002	-59.6
Management Level: Upper	.041	-0.6	.023	-38.0	.029	-13.1	.026	-12.0	.037	10.0
Management Level: Middle	.068	0.1	.035	-42.2	.042	-25.0	.037	-29.4	.033	-35.2
Management Level: Lower	.066	0.5	.026	-54.8	.03	-43.2	.031	-36.3	.049	-4.3
Management Level: None	.81	0.1	.912	9.7	.897	5.6	.905	4.6	.879	2.2
Part-time	.552	0.0	.589	6.9	.602	9.7	.606	10.3	.255	-20.7
Tenure	7.2	0.0	7.7	6.4	8.7	15.4	9.6	26.5	9.9	24.0
Industry: Low-tech manufacturing	.065	-0.5	.033	-44.4	.024	-54.6	.018	-59.6	.019	-51.2
Industry: High-tech manufacturing	.091	0.6	.064	-28.2	.068	-21.8	.062	-23.8	.069	-21.9
Industry: Less knowledge-intensive services	.376	-0.2	.473	23.0	.6	49.3	.661	55.4	.683	29.4
Industry: Knowledge-intensive services	.454	0.1	.424	-6.8	.305	-32.1	.256	-41.8	.226	-33.5
Industry: Other (incl. construction)	.014	1.4	.006	-48.1	.004	-60.4	.003	-63.5	.004	-41.4
Firm size: $¡$ 20	.074	-0.4	.015	-74.4	.006	-80.8	.005	-76.2	.003	-63.1
Firm size: 20–49	.065	0.5	.015	-72.0	.004	-87.9	.003	-86.2	.003	-74.3
Firm size: 50–249	.247	0.1	.211	-14.9	.101	-56.5	.071	-66.3	.042	-63.1
Firm size: 250–999	.203	0.0	.166	-18.9	.099	-51.9	.076	-60.8	.075	-51.1
Firm size: $\geq$ 1000	.411	-0.1	.593	36.0	.79	59.6	.845	53.1	.877	23.1
Occupation: Managers	.076	0.2	.036	-47.5	.049	-22.6	.048	-18.8	.078	3.7
Occupation: Professionals	.132	-0.1	.087	-33.1	.078	-37.8	.076	-35.1	.1	-10.6
Occupation: Technicians and Associate Professionals	.261	0.2	.228	-14.4	.154	-41.6	.112	-55.8	.098	-49.1
Occupation: Clerical Support Workers	.109	0.2	.056	-39.6	.085	14.3	.115	77.4	.199	118.8
Occupation: Services and Sales Workers	.269	0.0	.451	58.2	.447	40.9	.443	26.9	.351	6.5
Occupation: Craft and Related Trades Workers	.029	0.7	.017	-40.9	.016	-43.2	.012	-54.7	.012	-51.5
Occupation: Plant/Machine Operators, Assemblers	.022	1.4	.015	-36.8	.013	-45.6	.01	-50.1	.013	-42.9
Occupation: Elementary Occupations	.101	-0.2	.11	6.4	.16	51.9	.183	69.4	.149	-2.5
# Observed Women	4334		1579		722		331		35	

Notes: The table reports mean characteristics of women across support versions 1 to 5 (see Table 4). Mean values are calculated as averages over 1,000 randomly drawn subsamples of 10,000 observations from the full sample. In expectation, subsample means would match the full sample means if the support did not deteriorate more rapidly in smaller subsamples. Percentage differences (% Diff.) are relative to the mean characteristics in the full sample with the same support version.

References

Belloni, A., V. Chernozhukov, and C. Hansen (2013). Inference on Treatment Effects After Selection Amongst High-Dimensional Controls (with an Application to Abortion and Crime). *Review of Economic Studies 81(2)*, 608–650.

Belloni, A., V. Chernozhukov, and C. Hansen (2014). High-Dimensional Methods and Inference on Treatment and Structural Effects in Economics. *Journal of Economic Perspectives 28(2)*, 29–50.

Chernozhukov, V., D. Chetverikov, M. Demirer, E. Duflo, C. Hansen, W. Newey, and J. Robins (2018). Double/Debiased Machine Learning for Treatment and Structural Parameters. *Econometrics Journal 21(1)*, C1–C68.

Chetverikov, D., Z. Liao, and V. Chernozhukov (2021). On Cross-Validated Lasso in High-Dimensions. *Annals of Statistics 49(3)*, 1300–1317.

Hastie, T., R. Tibshirani, and J. Friedman (2009). *Elemants of Statistical Learning: Data Mining, Inference, and Prediction* (2nd ed.). Springer.

Hastie, T., R. Tibshirani, and M. Wainwright (2016). *Statistical Learning with Sparsity: The Lasso and Generalizations.* CRC Press.

Künzel, S., J. Sekhon, P. Bickel, and B. Yu (2019). Metalearners for Estimating Heterogeneous Treatment Effects using Machine Learning. *Proceedings in the National Academy of Science (PNAS) 116(10)*, 4156–4165.

Ñopo, H. (2008). Matching as a Tool to Decompose Wage Gaps. *Review of Economics and Statistics 90(2)*, 290–299.
